# Metabolic functions of *Pseudomonas fluorescens* strains from *Populus deltoides* depend on rhizosphere or endosphere isolation compartment

**DOI:** 10.3389/fmicb.2015.01118

**Published:** 2015-10-14

**Authors:** Collin M. Timm, Alisha G. Campbell, Sagar M. Utturkar, Se-Ran Jun, Rebecca E. Parales, Watumesa A. Tan, Michael S. Robeson, Tse-Yuan S. Lu, Sara Jawdy, Steven D. Brown, David W. Ussery, Christopher W. Schadt, Gerald A. Tuskan, Mitchel J. Doktycz, David J. Weston, Dale A. Pelletier

**Affiliations:** ^1^Biosciences Division, Oak Ridge National LaboratoryOak Ridge, TN, USA; ^2^Department of Natural Sciences, Northwest Missouri State UniversityMaryville, MO, USA; ^3^Graduate School of Genome Science and Technology, University of Tennessee, KnoxvilleKnoxville, TN, USA; ^4^Joint Institute for Computational Sciences, University of Tennessee, KnoxvilleKnoxville, TN, USA; ^5^Microbiology and Molecular Genetics, University of California, DavisDavis, CA, USA; ^6^Fish, Wildlife and Conservation Biology, Colorado State UniversityFort Collins, CO, USA; ^7^Department of Microbiology, University of Tennessee, KnoxvilleKnoxville, TN, USA

**Keywords:** microbiome, *Populus*, metabolism, endosphere, rhizosphere, metabolic modeling

## Abstract

The bacterial microbiota of plants is diverse, with 1000s of operational taxonomic units (OTUs) associated with any individual plant. In this work, we used phenotypic analysis, comparative genomics, and metabolic models to investigate the differences between 19 sequenced *Pseudomonas fluorescens* strains. These isolates represent a single OTU and were collected from the rhizosphere and endosphere of *Populus deltoides*. While no traits were exclusive to either endosphere or rhizosphere *P. fluorescens* isolates, multiple pathways relevant for plant-bacterial interactions are enriched in endosphere isolate genomes. Further, growth phenotypes such as phosphate solubilization, protease activity, denitrification and root growth promotion are biased toward endosphere isolates. Endosphere isolates have significantly more metabolic pathways for plant signaling compounds and an increased metabolic range that includes utilization of energy rich nucleotides and sugars, consistent with endosphere colonization. Rhizosphere *P. fluorescens* have fewer pathways representative of plant-bacterial interactions but show metabolic bias toward chemical substrates often found in root exudates. This work reveals the diverse functions that may contribute to colonization of the endosphere by bacteria and are enriched among closely related isolates.

## Introduction

In carbon-poor soil environments plant root exudates and fine root turnover provide a rich source of carbon substrates that attract and feed a plethora of soil bacteria (Bais et al., 2006). Plant-associated bacteria are diverse, with 50–1000 operational taxonomic units (OTUs) associated with any individual plant (DeAngelis et al., 2009; Uroz et al., 2010; Gottel et al., 2011; Weinert et al., 2011; Lundberg et al., 2012). While it is clear there is extreme phylogenetic diversity in the bacterial community, the functional diversity of bacteria and their contribution to the overall function of the microbiome is less apparent.

The root microbiota is commonly distinguished by two environments: the rhizosphere, the volume of soil directly influenced by the root, and the endosphere, the internal root tissue. The rhizosphere is generated by plant cell death and abscission from growing roots and/or active secretion of root exudate, a mixture of small molecules that can solubilize nutrients in the soil for subsequent uptake by the plant (Kirk et al., 1999; Dakora and Phillips, 2002). The specific chemical composition of the exudate depends on plant species, nutrient status (Dechassa and Schenk, 2004), environmental factors (Raynaud, 2010) and root age (Schnepf et al., 2012; Dunbabin et al., 2013), but generally has been shown to include amino acids and peptides, sugars, and small organic acids (Dakora and Phillips, 2002; Dechassa and Schenk, 2004; Carvalhais et al., 2011, 2013) that directly influence the microbial community associated with the plant (Glick, 2005; Bais et al., 2006; Hartmann et al., 2008; Stearns et al., 2012; Hunter et al., 2014; Ludwig-Müller, 2015). A relatively small fraction of bacteria that associate with the plant gain access to the internal root endosphere compartment (Compant et al., 2010; Gottel et al., 2011; Lundberg et al., 2012; Bulgarelli et al., 2013; Oldroyd, 2013; Shakya et al., 2013). These bacteria are exposed to a different biochemical environment which can include storage carbohydrates, complex structural polymers, and secondary metabolites such as nucleosides and aromatic compounds. Within the endosphere, bacteria can inhabit multiple environments such as inter- and intracellular spaces that may have a unique biochemical profile (Gaiero et al., 2013).

Relationships between bacteria and host plants, regardless of whether they are found in the rhizosphere or endosphere, can be mutually beneficial and enhance growth of both organisms. For example, plants in need of phosphorus exude organic acids to release soil-bound phosphates; the soil bacteria consume the organic acids from the plant and further solubilize phosphate in the environment, leading to increased available nutrient pools for both host and microbiome (Rodriguez et al., 2004; Vyas and Gulati, 2009; Ahemad and Khan, 2010). Beneficial bacteria can also induce systemic resistance in host plants to help prevent infection (Weston et al., 2012) or may directly inhibit pathogen growth through niche space competition or the production of antibiotics (Pérez-García et al., 2011). To thrive in the root microbiome, bacteria must compete with other community members for resources.

Investigation of the *Populus* rhizosphere microbiota by cultivation independent approaches has demonstrated that γ-*Proteobacteria*, primarily *Pseudomonas fluorescens*-like strains, are highly abundant and represent one of the dominant bacterial groups in this environment, along with α-*Proteobacteria, Acidobacteria*, and *Actinobacteria* (Gottel et al., 2011; Shakya et al., 2013). The *P. fluorescens* group includes many plant-associated strains and is genetically diverse (Silby et al., 2009; Loper et al., 2012), with recent assessments showing the core genome of 2789 genes (CDSs) only contributing ~50% to any individual genome in the group and a large pan-genome of 13,872 genes (Loper et al., 2012). Given the genetic diversity of *P. fluorescens*, it has been proposed that the group represents multiple bacterial species, however the boundaries between these species are often obscure (Silby et al., 2009; Loper et al., 2012). *Pseudomonas* species are well-studied for aerobic degradation of aromatic compounds (Stanier and Hayaishi, 1951; Díaz et al., 2013), a class of molecules that are prevalent in the *Populus* metabolome.

To investigate host-associated bacterial functional diversity rather than diversity driven by phylogeny or geographic location, we isolated diverse bacterial strains from the endosphere and rhizosphere compartments of native *Populus deltoides* trees in central Tennessee (Gottel et al., 2011; Weston et al., 2012). The observed diversity in the *Pseudomonas fluorescens* group (Silby et al., 2009; Loper et al., 2012) and the prevalence of *Pseudomonas* in our culture collection motivated us to investigate how genomic diversity and functional plasticity differ in endosphere and rhizosphere isolates collected from a single host plant species. Therefore, we have sequenced the genomes of 19 *Pseudomonas fluorescens* strains that are classified in the same OTU at 99% similarity by 16S rRNA gene sequencing from both the endosphere and rhizosphere compartments of *P. deltoides* roots (Brown et al., 2012). We screened these strains for functional attributes relevant to interaction with the host plant, including phosphate solubilization, denitrification, and ability to promote *Arabidopsis* root growth. Using both genomic and phenotypic analysis of the strains, we describe the diversity in these strains and identify attributes that distinguish strains isolated from the endosphere and rhizosphere. This work reveals the functional diversity that can exist within a single bacterial OTU in plant-microbiota systems, highlighting the complex associations between bacteria and their host organism.

## Methods

### Strain isolation

Strains were isolated from *Populus deltoides* roots collected in central Tennessee as described previously (Brown et al., 2012). Root samples were collected from mature *Populus deltoides* trees (36° 6′N, 85° 50′W, Supplemental File) in October 2009 near the Caney Fork River in the Buffalo Valley Recreation Area within DeKalb County, TN. Root samples were processed as described previously (Gottel et al., 2011; Weston et al., 2012). Rhizosphere strains were isolated by plating serial dilutions of root wash. Endosphere strains were isolated by pulverizing surface sterilized roots with a sterile mortar and pestle in 10 ml of MgSO_4_ (10 mM) solution and plating serial dilutions. The surface sterilization protocol is 5X washes with sterile water, followed by 30 s incubation in 95% ethanol, 3 min incubation in 5% NaOCl, then 6 washes with sterile water (Gottel et al., 2011). Strains were isolated on R2A agar media, and resulting colonies were picked and restreaked a minimum of three times to ensure isolation. Isolated strains were identified by 16S rDNA PCR amplification and sequence analysis.

### Genome analysis

Draft genome sequences for the 19 strains discussed in this study were used for all analyses and are publicly available in IMG (img.jgi.doe.gov) (Brown et al., 2012) and the genome assemblies for GM30, GM41 and GM80 have been improved (Utturkar et al., 2014). Sequencing, genome assembly, and genome annotation were described previously (Brown et al., 2012; Utturkar et al., 2014). The 16S rRNA consensus sequence was generated by aligning 16S rRNA genes from each genome and selecting the most frequently observed base as the consensus. Strains were then individually aligned to consensus and similarity was scored as the ratio of number of nucleotide differences to total nucleotides in the gene. Partitioned amino-acid sequence alignments of 10 genes common to all isolates (*acsA, aroE, dnaE, guaA, gyrB, mutL, ppsA, pyrE, recA*, and *rpoB*) was used for phylogenetic reconstruction via MrBayes (Ronquist et al., 2012). Predicted proteins from all isolates in this study and reference strain *P. fluorescens* Pf0-1, *P. fluorescens* SBW25, *P. protegens* Pf5. *P. putida* KT2440, *P. aeruginosa* PAO1, *P. syringae* strains DC3000, 1448a, and B728a were analyzed using OrthoMCL (Fischer et al., 2011) in order to assign the proteins to orthologous clusters. The default *e*-value of 1e-5 was used as a cutoff for inclusion into a cluster, and no cutoffs were used for percent identity or percent match. Genes present in all organisms were defined as core genes, and the remaining pan-genes were distributed across genomes. Manual curation of genomes was performed using IMG (img.jgi.doe.gov).

### Metabolic modeling

Metabolic models were generated from genome sequences for individual strains using the publicly-available KBase “Reconstruct Genome-Scale Metabolic Model” workflow with default parameters (kbase.us) [The Department of Energy Systems Biology Knowledgebase (KBase)]^1^. Models were gapfilled based on positive oxidation results for D-glucose and leucine. Transporters for sole carbon sources that tested positive for growth were added using KBase, then models were tested for ability to utilize carbon sources and scored for accuracy against experimental data.

### Physiological assays

Bacterial strains were maintained using R2A liquid or agar medium. Siderophore production was assayed in plate format using chrome azurol-S assay (Alexander and Zuberer, 1991). Protease activity was measured on skim milk agar plates (Sokol et al., 1979) and calcium phosphate solubilizing activity was tested using CaPhos plates (Katznelson and Bose, 1959). Denitrification activity was determined by growing strains anaerobically in stoppered tubes in the presence of 10 mM nitrate (or control) in R2A media for 3 days. Denitrification activity was determined by increase in optical density (660 nm). *Arabidopsis* phenotype was determined by transferring *A. thaliana* Col-0 seedlings to agar plates [1X Murashige and Skoog salts (Phytotechnology Laboratories) + 1% sucrose (wt/vol) (Sigma Aldrich) + 0.5 g/l MES salts (Sigma Aldrich) 0.7% Phytagar (Phytotechnology Laboratories)] and then streaking ~1 cm below roots with test strain. Phenotype was assessed visually after 14 days and compared to un-inoculated controls (Weston et al., 2012). Indole-3-acetic acid (IAA) concentrations in culture supernatants was determined by the colorimetric method of Salkowski (Glickmann and Dessaux, 1995): cells were grown in R2A media containing tryptophan (200 μg/ml), an IAA precursor, overnight at 25°C. A 1 ml aliquot of overnight culture was pelleted and 0.2 ml supernatant was mixed with 0.8 ml Salkowski's reagent (300 ml concentrated H_2_SO_4_, 2.03 g FeCl_3_-6H_2_O and 500 ml distilled H_2_O) and incubated at RT for 20 min. Red color formation was quantified as the absorbance (540 nm) using a CARY 100 UV-visible spectrophotometer (Varian Instruments, CA). A standard curve was prepared from serial dilutions of a 5 mM IAA stock solution in R2A. For antimicrobial activity, 5 μl of overnight R2A culture of test strain was spotted on R2A agar plate containing a lawn of *Escherichia coli* K12, *Bacillus subtilis, Candida albicans* C938 or *Schizosaccharomyces pombe* 972 h and incubated at 25°C overnight. A positive resulted was recorded when by zone of inhibition was observed around test strain.

### Sole carbon source oxidation testing and compound classification

Carbon oxidation was tested for all *Pseudomonas fluorescens* isolates in this study using Biolog PM1 and PM2A MicroPlate™ carbon sources, which contain 190 potential carbon substrates. Briefly, cells were grown overnight in 15 ml glutamine glucose minimal medium (GGMM) (Worm et al., 2000) at 25°C with shaking. 1.5 mL of culture was centrifuged for 1 min at 5000 rpm to pellet cells, then supernatant was removed and cells were resuspended cells in 1.5 ml of GGMM (no carbon). Biolog plates were inoculated with cells (OD = 0.1, 100 μL/well) mixed with dye A (1X concentration), covered and placed into an Omnilog reader and dye reduction results were reported at 24 h for duplicates. Biolog PM1 and PM2 compounds were classified using MetaCyc classification groups. A full table of classified compounds is available as a Supplemental File. For growth curves on sole carbon sources, strains were grown overnight in M9 media with glucose then pelleted and washed twice in M9 media with no carbon source. M9 media with 0.02 M carbon source were inoculated with strains at a starting OD of 0.05 (final volume 400 μL), then grown for 48 h at 25°C with constant shaking reading OD every hour.

### Statistics

Multivariate contingency χ^2^ analyses were used to test for non-homogeneity in presence of phenotypes or pathways or utilization of compound groups between rhizosphere and endosphere isolates. Isolates were considered as random effects representative of all possible endosphere and rhizosphere *Pseudomonas* strains. All phenotypes (e.g., siderophore production, denitrification, etc.) were considered as fixed effects and represented specific response variables in the χ^2^ analyses. Tests were implemented in MS Excel (see Supplemental File for tests).

## Results

### Genome statistics and phylogeny of rhizosphere and endosphere *Pseudomonas fluorescens* isolates

**Figure 1:**
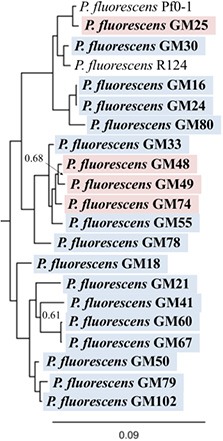
**Phylogenetic tree of ***P. fluorescens*** isolates**. Phylogenetic reconstruction based on the predicted protein sequences of 10 genes: *acsA, aroE, dnaE, guaA, gyrB, mutL, ppsA, pyrE, recA*, and *rpoB*. Red highlight, rhizosphere isolate, blue highlight: endosphere isolate. Two common reference isolates of *Pseudomonas* (Pf0-1 and R124) are shown for comparison. Full tree included in Figure S1. Scale bar is expected substitutions per site. Node labels indicate posterior probability, unlabeled nodes have values >0.99.

The 19 *Pseudomonas fluorescens* genomes range from 6.1 to 7.3 Mb and encode an average of 6076 genes. Genomic characterization and the location in the plant from which the strain was isolated are summarized in Table 1. Interestingly, both the functional prediction percentages and KEGG assignments were higher in rhizosphere isolates, indicating that endosphere isolates may encode uncharacterized pathways which contribute to the colonization of and interaction with the plant host. The full 16S rRNA gene sequences from these genomes are all at least 99% similar to the group consensus. To investigate the relationships further, a phylogenetic tree was generated based on the multi-locus sequencing approach (Ronquist et al., 2012) for 10 conserved genes recovered from the genomes. The resulting maximum-likelihood analysis also revealed, despite the use of 10 marker genes, a high degree of similarity (short branch lengths) between the rhizosphere and endosphere isolates (Figure 1 and Figure S1).

**Table 1:** **Genome statistics for ***P. fluorescens*** isolates**.

**Isolate**	**Isolation compartment**	**Genome size (Mb)**	**Scaffolds**	**GC (%)**	**Genes**	**Coding (%)**	**Functional prediction (%)**	**Assigned to KEGG (%)**	**Similarity to 16S consensus (%)**
GM25	Rhizosphere	6.37	186	61	5836	90.3	81.2	28.1	99.5
GM48	Rhizosphere	6.46	272	59	5977	87.7	81.1	29.1	99.6
GM49	Rhizosphere	6.60	370	60	6340	88.1	80.6	28.3	99.6
GM74	Rhizosphere	6.12	219	60	5649	88.2	81.4	29.8	99.6
GM16	Endosphere	6.56	155	59	5981	88.6	79.6	26.9	98.9
GM18	Endosphere	6.31	178	60	5797	89.2	80.5	28.8	99.3
GM21	Endosphere	6.62	258	58	6154	88.0	79.1	27.9	99.4
GM24	Endosphere	6.54	476	59	5974	88.8	79.3	27.0	99.3
GM30	Endosphere	6.15	215	60	5696	88.3	79.6	26.9	99.4
GM33	Endosphere	6.74	245	60	6177	88.3	82.0	28.6	99.3
GM41	Endosphere	6.63	230	59	6150	88.6	80.9	28.6	99.4
GM50	Endosphere	6.70	202	59	6151	88.1	79.9	27.5	99.2
GM55	Endosphere	6.50	217	60	6068	87.9	81.1	28.7	99.5
GM60	Endosphere	6.44	230	60	5991	88.2	80.9	28.5	99.6
GM67	Endosphere	6.52	254	60	6086	88.4	79.3	27.8	99.6
GM78	Endosphere	7.30	291	60	6804	88.8	81.0	27.7	99.6
GM79	Endosphere	6.72	166	59	6146	87.6	79.7	28.0	99.1
GM80	Endosphere	6.81	368	59	6334	88.2	77.2	25.8	99.4
GM102	Endosphere	6.67	207	59	6126	88.1	79.8	27.9	99.1

*Genome assembly statistics and functional predictions were extracted from annotated genomes using IMG (img.jgi.doe.gov). A consensus sequence of the 16S rRNA gene for all 19 strains was generated and used to compare all strains' 16S rRNA gene sequence*.

### Functional screening of plant interaction phenotypes

**Figure 2:**
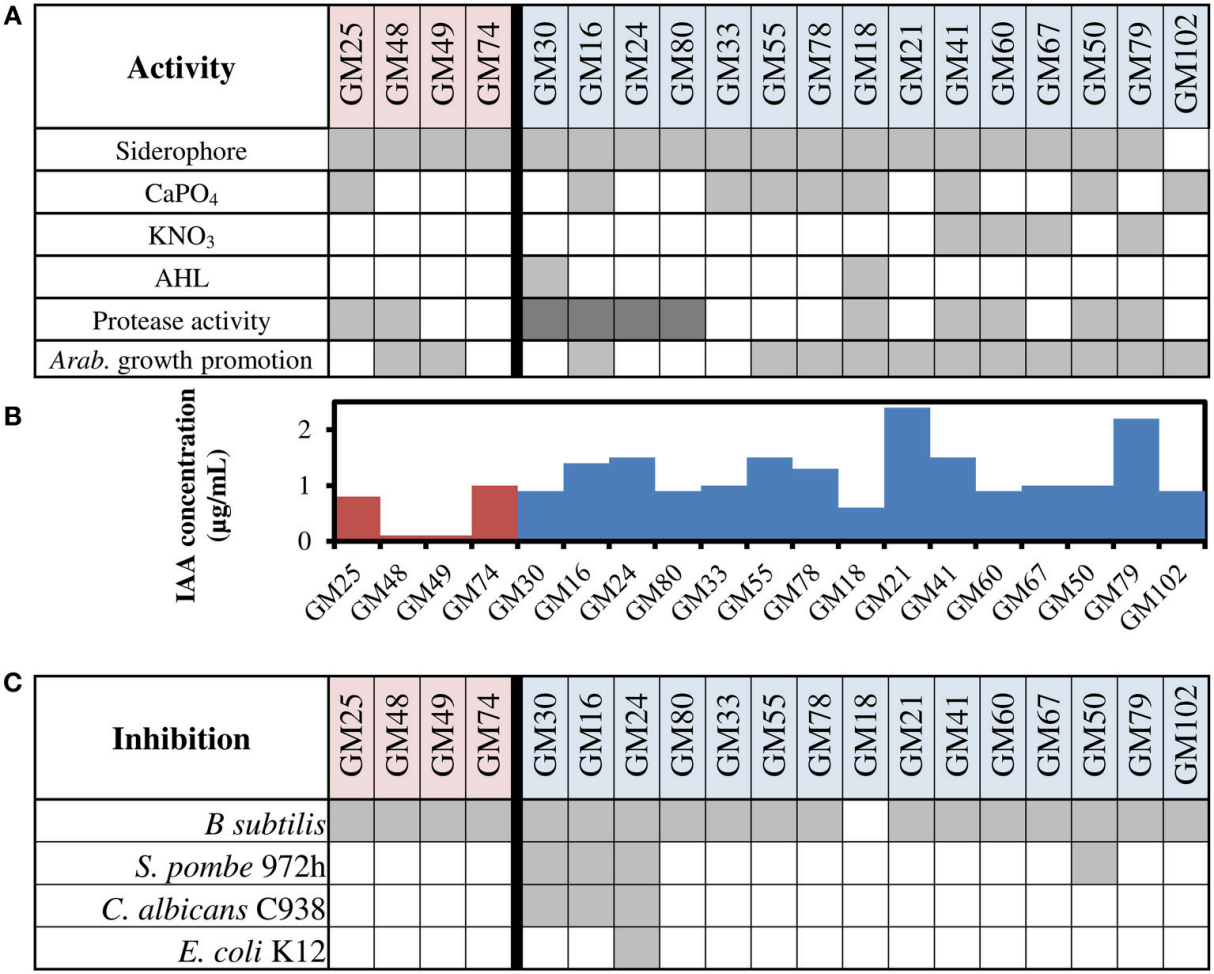
**Phenotype screening of ***P. fluorescens*** isolates. (A)** Siderophore production, solubilization of calcium phosphate (CaPO_4_), denitrification by anaerobic growth with potassium nitrate, acyl-homoserine lactone (AHL) production, protease activity, and growth promotion of *Arabidopsis* roots. Gray boxes indicate positive result of assay and white boxes indicate negative result of assay; dark gray indicates high activity for protease assay. Red shading on strain designation indicates rhizosphere isolate, blue shading indicates endosphere isolate **(B)** Production of indole-3-acetic acid production (IAA) using the Salkowski method. Red bars are results for rhizosphere isolates, and blue bars are results for endosphere isolates **(C)** Inhibition of four tested strains by lawn assay. Gray boxes indicate inhibition of strain by isolate.

The contribution of the microbiome to host plant phenotype is multi-functional and can occur through direct interactions via specific mechanisms, or indirect interactions through environmental modifications that mutually benefit the host, bacteria, and other community members. All *P. fluorescens* isolates in this study were tested for several activities relevant to plant-microbe interactions (Figure 2). For example, activities such as the production of siderophores (Poole and McKay, 2003), production of the plant hormone indole-3-acetic acid (IAA) (Di Simine et al., 1998; Sridevi and Mallaiah, 2009; Vyas and Gulati, 2009; Marra et al., 2012; Wang et al., 2012), and protease activity are characteristics common to rhizosphere and endosphere isolates of *P. fluorescens* (O'Sullivan and O'Gara, 1992).

Siderophore production was positive for all strains except strain GM102. Several strains were capable of calcium phosphate solubilization, a phenotype biased toward endosphere isolates (8/15) over rhizosphere isolates (1/4). Denitrification activity was limited to four endosphere isolates and was not observed in rhizosphere isolates.

The production of secreted proteases may impact the ability of bacteria to enter the endosphere or metabolize different substrates. The majority of endosphere isolates displayed protease activity, and four of the endosphere isolates displayed high levels of protease activity. Two rhizosphere isolates also showed protease activity, though the presence of the exo-protease chitinase was found within eight of fifteen genome sequences of endosphere isolates (GM16, 24, 30, 55, 67, 80, and 102) and only one genome sequence of the rhizosphere isolates (GM25).

All isolates were tested specifically for their ability to affect root growth using *Arabidopsis thaliana* seedlings (Supplemental File). All isolates increased root branching relative to controls. Most did not affect root length, but endosphere isolates GM24, GM30, and GM33 and rhizosphere isolates GM25 and GM74 decreased root lengths.

All isolates were capable of producing the plant growth promoting hormone indole-3-acetic acid (Spaepen et al., 2007; Santner and Estelle, 2009; Gallavotti, 2013; Pacifici et al., 2015). The average concentration produced by endosphere isolates was significantly higher than rhizosphere isolates (*p* < 0.01, Figure 2B). The *iaaH* and *iaaM* genes used for biosynthesis of IAA from tryptophan were found in GM16 and GM24, and both strains lack the gene encoding tryptophan 2,3-dioxygenase. Consistent with the relatively high production of IAA in GM16 and GM24, the absence of tryptophan 2,3-dioxygenase would ensure all excess tryptophan is used for IAA biosynthesis instead of tryptophan metabolism (Taghavi et al., 2009). Contrary to this assumption, the two isolates with the highest IAA production (>2 μg/ml, GM21 and 79) were not found to encode any tryptophan-derived, IAA biosynthesis pathways. Furthermore, the three lowest IAA levels measured were in isolates containing tryptophan 2, 3-dioxygenase (GM18, 48, 49) (0.6, 0.1, 0.1 μg/mL). Only isolate GM79 contained a gene encoding tryptophan 2, 3-dioxygenase, while still producing higher levels of IAA (1.3 μg/mL), suggesting that there may be other important pathways for IAA biosynthesis that are not yet understood.

The assessment of antimicrobial production revealed that the majority of isolates were able to inhibit at least one of four tested organisms (*Escherichia coli, Bacillus subtilis, Candida albicans* and *Schizosaccharomyces pombe*, Figure 2C). All strains except GM18 inhibited *B. subtilis*. In contrast, only GM24 inhibited the growth of all four organisms including *E. coli*.

### Pathway analysis reveals significant diversity and biases in endosphere and rhizosphere isolates

**Figure 3:**
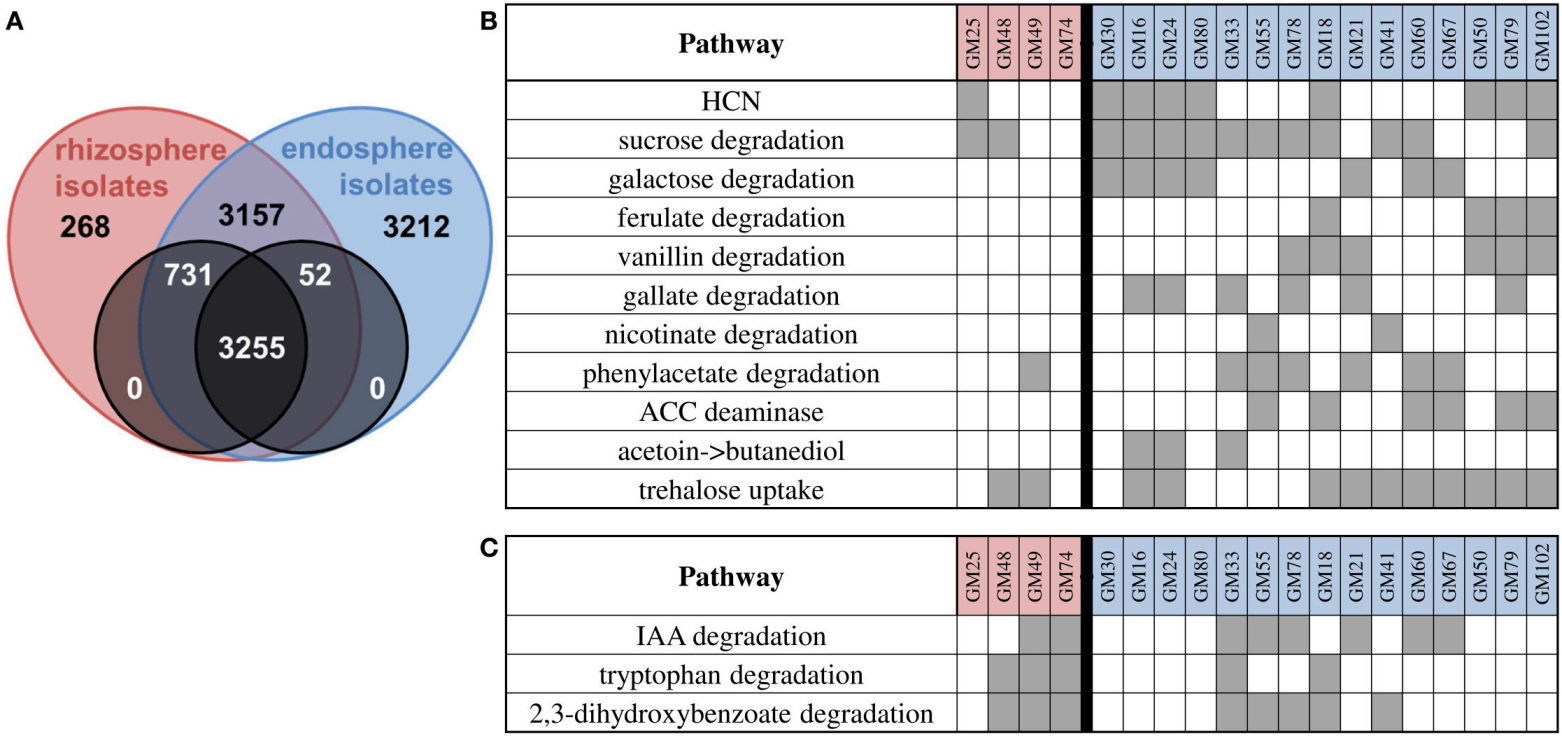
**Core/pan genome summary and pathway identification. (A)** Core/pan-analysis using ORTHOMCL clustering. There are 3255 genes shared between all isolates (black overlap), with 731 in all rhizosphere and some endosphere isolate genomes, and 52 in all endosphere and some rhizosphere isolate genomes. 3157 genes are shared between some rhizosphere and some endosphere isolate genomes. 268 and 3212 genes are shared between rhizosphere isolates or endosphere isolates only. The zeros indicate that there are no genes in all endosphere isolate genomes and no rhizosphere isolate genomes or vice versa. **(B)** Summary of manually identified pathways biased toward endosphere isolates. **(C)** Summary of manually identified pathways biased toward rhizosphere isolates. Gray boxes indicate presence of pathway in organisms.

The different environmental conditions between the rhizosphere and endosphere may necessitate the production of plant signaling compounds or degradation of metabolites abundant in either environment. Core/pan-genome analysis (using OrthoMCL clustering) revealed 3255 genes common to all strains and 2008 genes shared differentially between strains (Figure 3A). We observed more genes in the endosphere isolate pan genome (3212 genes in 15 genomes, 214 per genome) relative to the rhizosphere isolates (268 in 4 genomes, 67 per genome). Similarly there are more genes in the rhizosphere core that are represented in the endosphere isolates relative to endosphere core genes in rhizosphere isolates. These data suggest that endosphere isolates have additional genes relative to rhizosphere isolates, presumably contributing to their ability to persist in the endosphere. Interestingly, we did not observe genes that were unique to all endosphere or all rhizosphere isolates, indicating that in our data set no single metabolic function is correlated with competence in the endosphere or rhizosphere compartments for the strains in this study.

We performed manual curation to identify pathways encoded in genomes of endosphere or rhizosphere isolates (Figures 3B,C). Alone, none of the pathways distinguish endosphere from rhizosphere isolates but as a group the genomes of endosphere isolates genomes are enriched for pathways related to the production or degradation of plant metabolites and signaling molecules (multivariate contingency χ^2^ analysis, α = 0.05).

Genes for antibiotic production in the *Populus*-associated *P. fluorescens* strains were limited. None of the strains had genes necessary for 2, 4-diacetylphloroglucinol (DAPG) or pyoluteorin production. However, the genes required for hydrogen cyanide production were found in the majority of endosphere isolates (8/15) but only one rhizosphere isolate (GM25).

Corresponding to increased availability of metabolites such as storage carbohydrates in the root endosphere, a higher fraction of endosphere isolate genomes contain genes for carbohydrate degradation pathways. The majority of endosphere isolates (12/15) have sucrose degradation pathways while only 2/4 rhizosphere isolates have sucrose degradation pathways. Galactose degradation is also abundant in endosphere isolates (7/15) with no rhizosphere isolate containing this pathway.

*Pseudomonas* strains are known to degrade a wide range of aromatic plant metabolites (Stanier et al., 1966; Dewick and Haslam, 1969; Foyer et al., 2003; Cooke et al., 2005; Chen et al., 2009; Smith et al., 2011; Li et al., 2014). All strains in this study, except for GM30, carry the *benABCD*/*catBCA* clusters involved in benzoate catabolism and in the *ortho*-cleavage pathway of catechol, a common intermediate in a variety of aromatic compound degradation pathways (Harwood and Parales, 1996). All strains also possess the *pobRA* and *pcaRKIJFHGTBDC* clusters for the conversion of 4-hydroxybenzoate to protocatechuate and subsequently to TCA cycle intermediates (Jiménez et al., 2002). Phenylpropanoids such as ferulate, caffeate, and coumarate are a vast group of aromatic compounds that are synthesized by plants from the amino acid phenylalanine (Hahlbrock and Scheel, 1989). Endophyte isolates GM18, 50, 79, and 102 carry the *fcs/ech/vdh* genes adjacent to the *vanAB* genes, all of which are required for phenylpropanoid degradation via protocatechuate (Priefert et al., 1997; Overhage et al., 1999; Jiménez et al., 2002; Plaggenborg et al., 2003; Calisti et al., 2008; Havkin-Frenkel and Belanger, 2008). In addition, strains GM21 and 78 possess only the *vanAB* genes for vanillin catabolism. Ferulate and vanillin degradation pathways are not found in rhizosphere isolates.

A majority of endophyte strains (10/15) and one rhizosphere strain encode for the degradation of gallic acid, nicotinic acid, or phenylacetic acid, catabolic pathways that are not present in soil isolate *P. fluorescens* Pf0-1, suggesting that metabolism of these compounds is important for endophyte strains. Gallic acid (3,4,5-trihydrobenzoic acid) is a phenolic compound produced by plants (Dewick and Haslam, 1969) and the presence of *gal* cluster required for gallic acid degradation was found in strains GM16, 21, 24, 33, 78, and 79 with gene organization identical to that in *P. putida* KT2440 (Nogales et al., 2011). Strains GM41 and 55 possess the *nic* cluster required for degradation of nicotinic acid, a carboxylic acid derivative of pyridine that is widely distributed in the environment (Kaiser et al., 1996; Fetzner, 1998). The *pha* gene cluster, which encodes enzymes for phenylacetic acid catabolism (Jiménez et al., 2002), is present in several endosphere isolates (6/15) and one rhizosphere isolate (GM49).

The gene for 1-aminocyclopropane-1-carboxylate (ACC) deaminase was found in 6/15 endosphere isolates (GM18, 55, 60, 67, 79, 102) and 0/4 rhizosphere isolates. This enzyme has been shown to lower ethylene levels in plants and is a well-studied example in plant-microbe interactions (Bulgarelli et al., 2013). In addition, both acetoin and 2,3-butanediol can promote plant growth (Ryu et al., 2003). Endosphere isolates GM16, 24, 33, 41, and 50 have the *acoR* gene for transcriptional regulation, and in GM16, 24, and 33, this gene is near an acetoin reductase that can convert acetoin to 2,3-butanediol. One of these two pathways (ACC deaminase or 2,3-butanediol production) is present in 11/15 endosphere isolates and 0/4 rhizosphere isolates.

Analysis of transporter classes showed that endosphere isolates had significantly more efflux transporters than rhizosphere isolates (*p* = 0.002). Within this group, sugar, Ni^2+^, K^+^, heme and Fe^3+^ transporters were higher in endosphere isolates. Only a subset of our isolates contained the *treRBAP* and *lamB* gene neighborhood that is used for trehalose uptake and utilization. These genes are found in *P. protegens* Pf-5 and SBW25. Trehalose uptake was found in the majority of endosphere isolates (GM16, 18, 21, 24, 41, 50, 60, 67, 79, and 102) and GM48 and GM49, both rhizosphere isolates. Trehalose biosynthesis has been found to be important to the survival of *P. putida* in low-humidity soil (Roca et al., 2013) and all of our isolates have the genes necessary for production of trehalose from both maltose and maltodextrin.

Rhizosphere strains were biased toward different pathways for degradation of plant-produced metabolites (Figure 3C). Rhizosphere strains GM48, GM49, GM74 and endophyte strains GM18 and GM33 possess genes required for tryptophan catabolism. All strains with this pathway also appeared to have acquired a specialized porin for uptake of tryptophan encoded by *kynF*. There was also evidence within the genomes for IAA catabolism which could affect host plant or microbial-derived IAA signaling. Genes for the *iacHABICDEFG* gene neighborhood for IAA catabolism (Leveau and Gerards, 2008) were found in six endosphere isolates (GM21, 33, 55, 60, 67, 78) and two rhizosphere isolates (GM49, 74).

Homologs of the *cmtC* and *cmtD* genes from the *p*-cumate degradation pathway were identified, which encode for 2,3-dihydroxy-*p*-cumate dioxygenase and a decarboxylase, followed by a set of genes whose products feed the resulting degradation intermediate into the *meta*-cleavage pathway (Eaton, 1996) in 3/4 rhizosphere and 5/15 endosphere isolates. These genes are likely involved in degradation of the plant metabolite 2,3-dihydroxybenzoate as observed in *P. fluorescens* (DeFrank and Ribbons, 1977) and *P. reinekei* MT1 (Marín et al., 2012).

### Comparative metabolic model construction for *Pseudomonas* isolates reveals additional predicted metabolic capability in endosphere isolates

**Figure 4:**
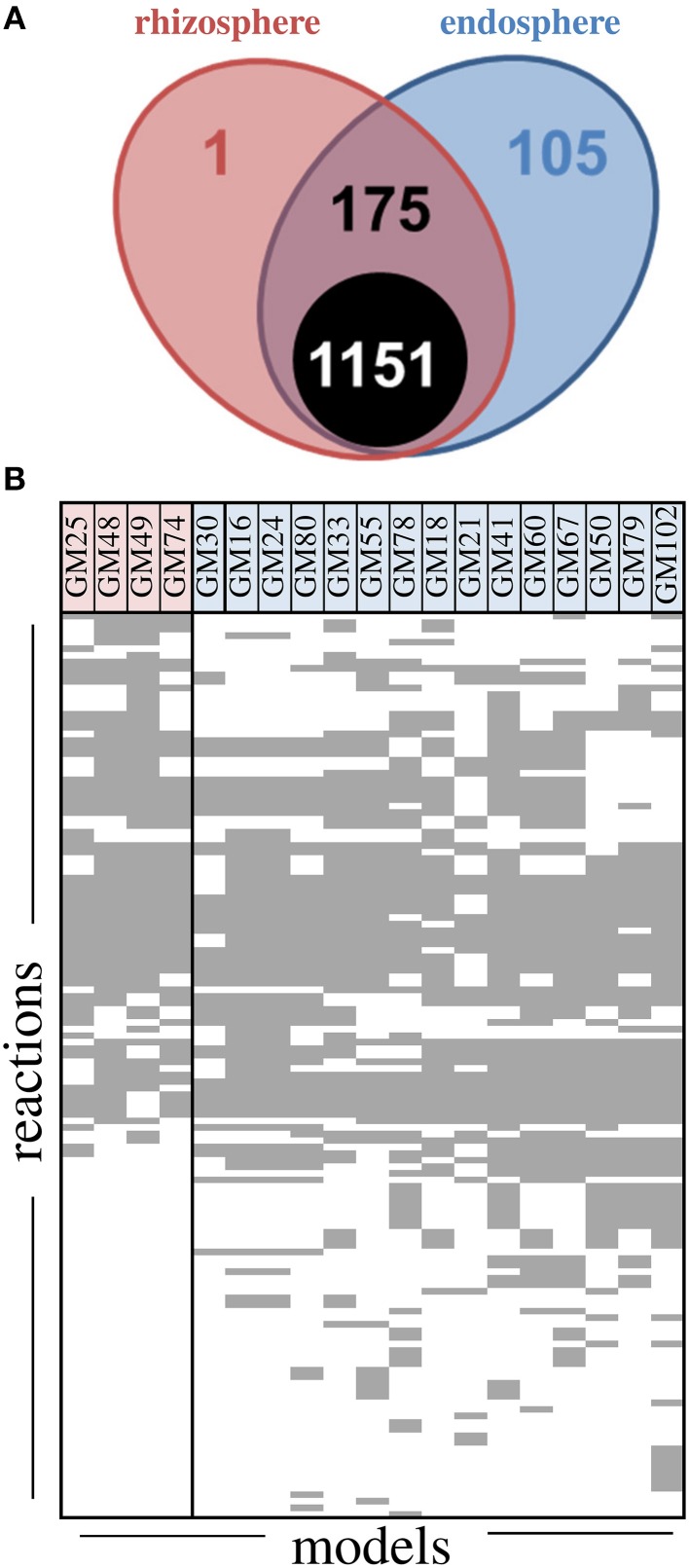
**Metabolic reconstruction summary for ***Pseudomonas fluorescens*** isolates. (A)** Number of reactions in models grouped by rhizosphere and endosphere. The black circle indicates the number of reactions common to all models, 175 reactions are shared between some endosphere and rhizosphere, and one reaction is unique to rhizosphere while 105 are unique to endosphere isolates. **(B)** Model summaries for non-core reactions. Each row represents a non-core reaction and each column represents the model from a single isolate.

The overall metabolic processes in strains were compared by using genome-scale metabolic reconstructions. Models were generated for each isolate using online tools (kbase.us) and ranged from 1235 to 1324 reactions with 1151 reactions common to all models (Figure 4). Of the 281 reactions distributed differentially throughout the models, 42 were predicted transporters and 61 were not classified in KEGG maps. In general the unclassified reactions were involved in the synthesis of fatty acids (Supplemental File). A large fraction of the differentially distributed reactions (105/281) were only found in endosphere isolates, while only one reaction was unique to rhizosphere isolates (transport of sodium and L-malate across cell membrane). The reactions involved in tryptophan and inositol metabolism and 2-oxopentenoate degradation were overrepresented in rhizosphere isolates while pyrimidine, ascorbate, aldarate, and phenylalanine metabolism, biosynthesis of lysine and folate, and degradation of methylsalicylate were overrepresented in endosphere isolates. The degradation of these groups is consistent with abundance of aromatic compounds in the *Populus* metabolome (Chen et al., 2009).

Overall model accuracy, when tested for prediction of sole carbon source utilization data, was 76%, with a maximum of 82% for strain GM30 and minimum of 67% for GM16, both endosphere isolates (Figure S2). The models predicted 28 compounds as core compounds that all strains should grow on as compared to the 47 core compounds observed in experimental data. Model prediction indicated that all strains except GM18 and GM74 used L-histidine and β-hydroxybutyric acid, respectively. A notable false negative was 4-hydroxybenzoate, a metabolite common in *Populus* leaves. That is, all strains grew on 4-hydroxybenzoate when tested, but none were predicted to grow based on automatically generated models, although the pathways were identified by manual curation. Overall, we observe additional metabolic reactions in endosphere models, suggesting additional metabolic capabilities in endosphere isolates relative to rhizosphere isolates.

### Carbon substrate utilization differs between endosphere and rhizosphere isolates

Given metabolic biases observed in models and manual genome analysis, strains were tested for their ability to oxidize sole carbon sources and results were analyzed for biases toward endosphere or rhizosphere isolates, as has been shown in previous studies for pathogenicity (Monk et al., 2013) or isolation environment (Malfanova et al., 2013). The number of compounds metabolized by endosphere isolates ranged from 74 to 94; rhizosphere isolates ranged from 72 to 82 out of 190 tested compounds (Biolog PM1 and PM2 plates). Within the endosphere group, 51 compounds were used by all isolates, 71 by none, and 68 were differentially used throughout the group. For rhizosphere isolates, 62 compounds were used by all isolates, 95 by none, and 33 were differentially used throughout the group (Figure S3). Between rhizosphere and endosphere groups, there were no compounds that were used by all of one group and none in the other. However, of all the compounds not used by rhizosphere isolates, at least one endosphere isolates was able to utilize at least one of those compounds. This pattern suggests that endosphere isolates have additional metabolic capabilities relative to rhizosphere isolates, consistent with the phenotype data and genomic analysis of metabolic ability. However, this result may reflect sampling bias due to the lower number of rhizosphere strains relative to endosphere strains.

**Figure 5:**
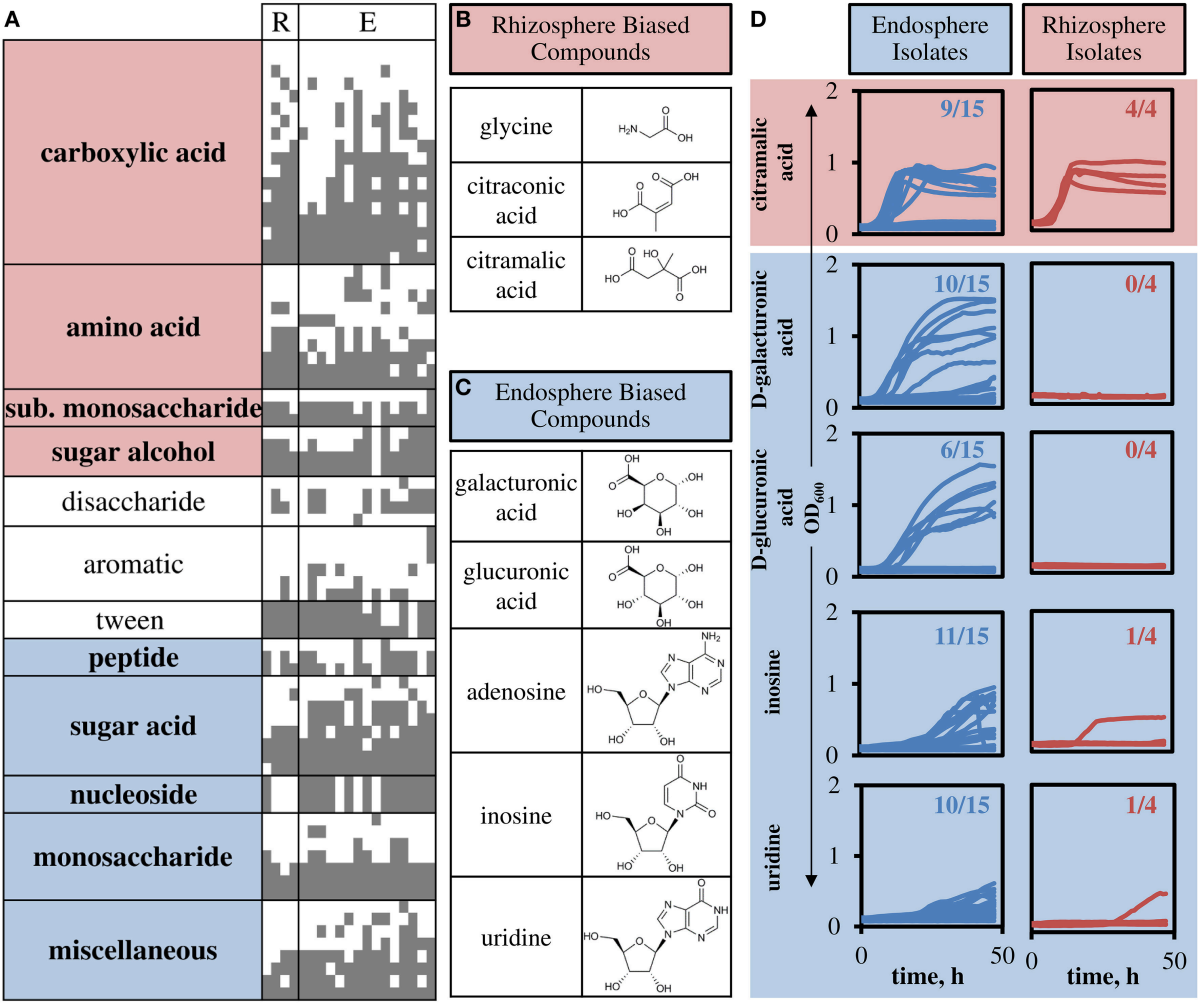
**Compound groups and individual substrates that differentiate endosphere and rhizosphere isolates. (A)** Carbon sources are listed vertically (compound group shown), and strains are listed horizontally (R, rhizosphere strains; E, endosphere strains). A gray square indicates metabolic activity in the presence of the substrate. Only differentially used substrates are shown, full table with specific compounds is included in Supplemental File. Highlighting on compound groups indicates significant bias toward rhizosphere (red) or endosphere (blue) isolates. **(B)** Three compounds that showed highest rhizosphere bias. **(C)** Five compounds that showed highest endosphere bias. **(D)** Growth curves for sole carbon sources with observed growth in M9 minimal media. Numbers represent the number of isolates that were shown to grow using the specified compound as the sole carbon source.

The 190 tested compounds were grouped into classes based on functional side groups (Supplemental File), and then groups were tested for non-homogeneity in substrate oxidation biased toward rhizosphere or endosphere isolates. Nearly all groups displayed non-homogeneity in substrate oxidation biases (χ^2^, α ≤ 0.01, Figure 5). Heterogeneous groups were classified as rhizosphere or endosphere biased by calculating fraction of isolates which oxidized substrates in the group. That is, carboxylic acids, amino acids, substituted monosaccharide and sugar alcohol groups were biased toward rhizosphere isolates, consistent with exudate profiles in plants. Compound groups biased toward endosphere isolates included complex substrate groups of peptides, sugar acids, nucleosides, and monosaccharides, compounds abundant in the endosphere environment. The miscellaneous group of compounds was also biased toward endosphere isolates.

Based on the identified groups, we chose representative compounds that were highly biased toward utilization by rhizosphere or endosphere isolates to confirm growth (Figures 5B,C). All isolates were tested for growth on three metabolites biased toward utilization by rhizosphere isolates and five biased toward utilization by endosphere isolates. Glycine and citraconic acid did not show growth by these isolates in M9 minimal media (data not shown), but citramalic acid corroborated the carbon oxidation panel results, such that, all rhizosphere isolates grew on citramalic acid and nine endosphere isolates also grew on citramalic acid (Figure 5D, Figure S4). Of the five endosphere biased metabolites, only adenosine showed no growth in the growth assay (data not shown). The remaining four showed growth of most endosphere isolates and one or none of the rhizosphere isolates (Figure 5D, Figure S4).

## Discussion

In this study we compared genome sequences and phenotypes of 19 *Pseudomonas fluorescens* strains isolated from the *Populus deltoides* endosphere or rhizosphere. Despite the similar isolation conditions and relative taxonomic closeness of these isolates (99% similarity between 16S rRNA genes), there was significant diversity in the genomes and phenotypes, highlighting the considerable functional diversity that can exist within a single OTU class in the plant microbiome. There were no gene clusters or phenotypic traits that uniquely discriminated between rhizosphere and endosphere isolates, which could be attributed to the: (1) wide range of potential mechanisms for plant-bacteria interactions, (2) misidentification of pathways, (3) actual expression of these pathways on plant, or (4) inability to predict function for all genes. However, within the strains isolated from endosphere or rhizosphere, we observed trends that require further study. In endosphere isolates we observed additional genomic elements dedicated to the metabolism of plant-relevant compounds, e.g., either synthesis or modification of plant hormones or catabolism of nucleosides and sugar acids, carbon-rich and complex molecules, which are more abundant in the endosphere compartment. The most distinguishing plant-relevant phenotypes were production of IAA, antimicrobial compounds and denitrification, all of which were biased toward endosphere isolates. The production of IAA has been observed in numerous plant growth promoting bacteria (Spaepen et al., 2007; Santner and Estelle, 2009; Gallavotti, 2013; Pacifici et al., 2015).

Phenotype data showed that endosphere isolates could perform more activities relevant to interactions with the plant or competition in the microbiome relative to rhizosphere isolates. That is, the measured activities contribute to overall system function by direct interaction through molecular signaling or by indirect mechanisms due to changes in microbiome composition or nutrient availability. Nearly all isolates showed antimicrobial activity as measured by the ability to inhibit growth of four test organisms, but endosphere isolates generally inhibited a higher proportion of the tested organisms. There is more phylogenetic diversity in the rhizosphere (Bulgarelli et al., 2013), suggesting more interspecific competition, and potentially necessitating the ability to inhibit a broader range of organisms, but our activity results did not support this hypothesis. Alternatively, the production of anti-microbial compounds and inhibition of growth within the endosphere can contribute to pathogen resistance (Mazzola et al., 2014; De Coninck et al., 2015) or biocontrol of the community (Vetsigian et al., 2011; Tyc et al., 2015), both mechanisms ultimately benefiting the host plant. Four endosphere isolates were capable of denitrification, which has been shown to be a beneficial function for competitive ability for *P. fluorescens* in the rhizosphere (Ghiglione et al., 2002) and for colonization in the endosphere in *Ralstonia* infections of plants (Dalsing et al., 2015), likely due to the growth advantage in micro-aerobic environments in the endosphere due to the ability to use nitrate as an electron acceptor.

Endosphere isolates tended to have additional pathways relative to rhizosphere isolates, as indicated by pan-genome analysis, metabolic models, and manual pathway identification. Unexpectedly, we did not observe relatively smaller genome sizes in endosphere isolate indicative of evolution of symbiotic relationships (McCutcheon and Moran, 2011). In fact, the endosphere isolates appeared to have relatively larger genome sizes relative to rhizosphere isolates, potentially due to a requirement that endosphere isolates must provide some benefit to the host, while still being able to survive and compete in the soil environment. In rhizosphere isolates we observed genomic biases toward cell structure biosynthesis, cofactor production pathways, and metabolism of amino acids and carboxylic acids, consistent with adaptation to an environment with less nutrient availability. Alternatively, endosphere isolates have access to complex cofactors and are under less pressure to maintain diverse, alternate pathways. For example, tryptophan catabolism via the kynurenine pathway proceeds by converting L-tryptophan into anthranilate, which is processed into catechol before entering the *ortho*-cleavage pathway (Stanier and Hayaishi, 1951; Koushik et al., 1997; Kurnasov et al., 2003). Anthranilate can also be siphoned into the biosynthesis of nicotinamide adenine dinucleotide (NAD) and quinolones (Farrow and Pesci, 2007), potentially important for growth in carbon-poor environments. Another explanation for increased genome size stems from the decreased diversity in the endosphere relative to rhizosphere, such that, the strains that do have access to the endosphere may have to make up for the lack of diversity by performing the anti-microbial duties that are performed by other community members in the rhizosphere.

The availability of specific carbon sources is a strong selection for bacterial adaptation. The results of this study show that classes of molecules rather than specific metabolites distinguish endosphere isolates from rhizosphere isolates. Specifically, endosphere isolates were biased toward the catabolism of peptides, sugar acids, nucleosides and monosaccharides, compounds that are expected to be prevalent in the endosphere. One of the highly biased compounds (10/14 endosphere, 0/4 rhizosphere isolates), galacturonic acid, is the monomer found in pectin, a polysaccharide commonly found in plants and reported in *Populus* roots (Cooke et al., 2005; Smith et al., 2011). Rhizosphere isolates were biased toward carboxylic and amino acids, substituted monosaccharides and sugar alcohols, compounds potentially prevalent in root exudates. It is unclear how the consumption of plant-produced carbon sources by bacteria directly impacts the host, though carbon source has been shown to dictate *Enterobacter* gene expression thus serving as a signal for interaction with the host plant (Taghavi et al., 2015).

It is likely that the definitions of endosphere and rhizosphere in this study are too coarse to attribute to specific phenotypes. Within the endosphere, strains can colonize multiple root tissues and may be localized to the inter- or intra-cellular space within those tissues. The endosphere is not chemically homogeneous and may have specific zones such as root tips, branch points, or structural components that have alternate chemical compositions/environments. Similarly, the rhizosphere is spatially heterogeneous. Energy rich compounds secreted by the root are most likely degraded rapidly by rhizosphere bacteria, while lower energy compounds could persist and diffuse farther from the root, generating a gradient that could impact rhizosphere bacteria. Further, the rhizosphere chemical composition at root hairs is different than the chemical composition at the root tip due to programmed cell death and cell abscission at the root tip during active growth. These spatial heterogeneities define niches to which specific bacteria can adapt. All of these examples would be masked by the current definition of endosphere and rhizosphere.

Similar to previous studies of the *Pseudomonas fluorescens* group (Silby et al., 2009; Loper et al., 2012), we also observed three clades within our genomes, supporting the segregation of the *P. fluorescens* group into multiple species. Despite the potential speciation, we observe functional ability (both genomic and phenotypic) correlated with isolation compartment, highlighting potential functional requirements for colonization of the endosphere or rhizosphere environments. The diversity in functions displayed by the isolates in this study suggests that bacteria from a single OTU can fill multiple roles in the microbiome, potentially explaining the poor correlation between host genotype and microbiome as measured at the OTU level (Shakya et al., 2013).

## Author's note

This manuscript has been authored by UT-Battelle, LLC under Contract No. DE-AC05-00OR22725 with the U.S. Department of Energy. The United States Government retains and the publisher, by accepting the article for publication, acknowledges that the United States Government retains a non-exclusive, paid-up, irrevocable, world-wide license to publish or reproduce the published form of this manuscript, or allow others to do so, for United States Government purposes. The Department of Energy will provide public access to these results of federally sponsored research in accordance with the DOE Public Access Plan (http://energy.gov/downloads/doe-public-access-plan).

### Conflict of interest statement

The authors declare that the research was conducted in the absence of any commercial or financial relationships that could be construed as a potential conflict of interest.

## Footnotes

^1^The Department of Energy Systems Biology Knowledgebase (KBase). Available online at: https://kbase.us.
