# Supplementary material for: Metabolic functions of Pseudomonas fluorescens strains from Populus deltoides depend on rhizosphere or endosphere isolation compartment
Source: Front Microbiol. 2015 Oct 14;6:1118. doi: 10.3389/fmicb.2015.01118 (PMC4604316; doi:10.3389/fmicb.2015.01118)
Supplement: Supplementary file 1 [file Presentation1.PDF]

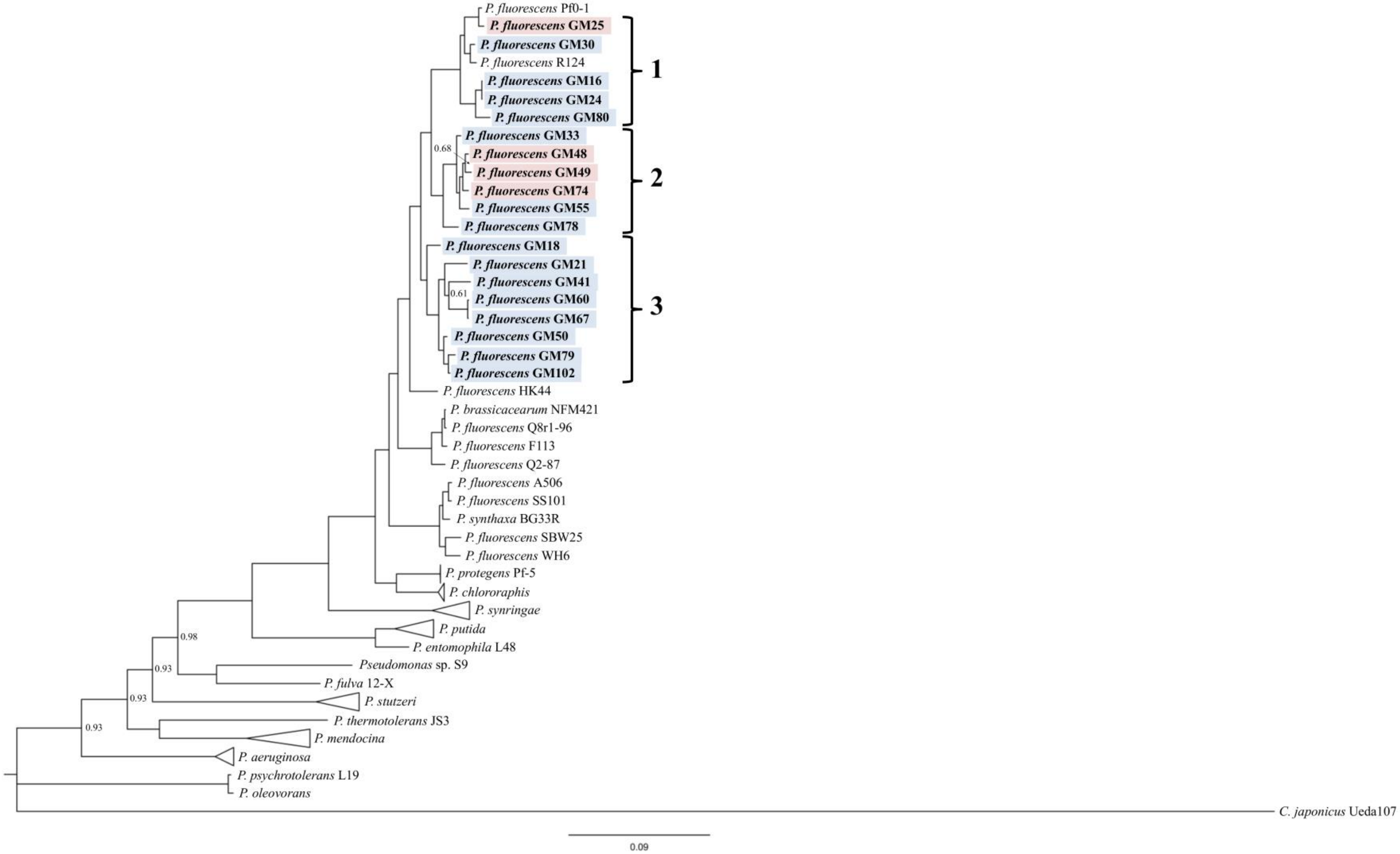


**Figure S1. Phylogenetic analysis of 21 *Populus* microbiome *Pseudomonas* isolates and related *Pseudomonas* species.** Phylogeny is based on a concatenated alignment of 10 housekeeping genes: *acsA*, *aroE*, *dnaE*, *guaA*, *gyrB*, *mutL*, *ppsA*, *pyrE*, *recA* and *rpoB*, and analyzed as separate gene partitions within MrBayes (Ronquist *et al*. 2012). The best model of protein evolution was chosen for each gene partition via ProtTest (Darriba *et al*. 2011). The closest appropriate model allowable within MrBayes was used and each partition was further allowed to vary independently. Isolates from this study are shown in bold. The majority of isolates formed three distinct subgroups that have been labeled 1, 2 and 3. Scale bar is expected substitutions per site. Node labels indicate posterior probability, unlabeled nodes have values >0.99.

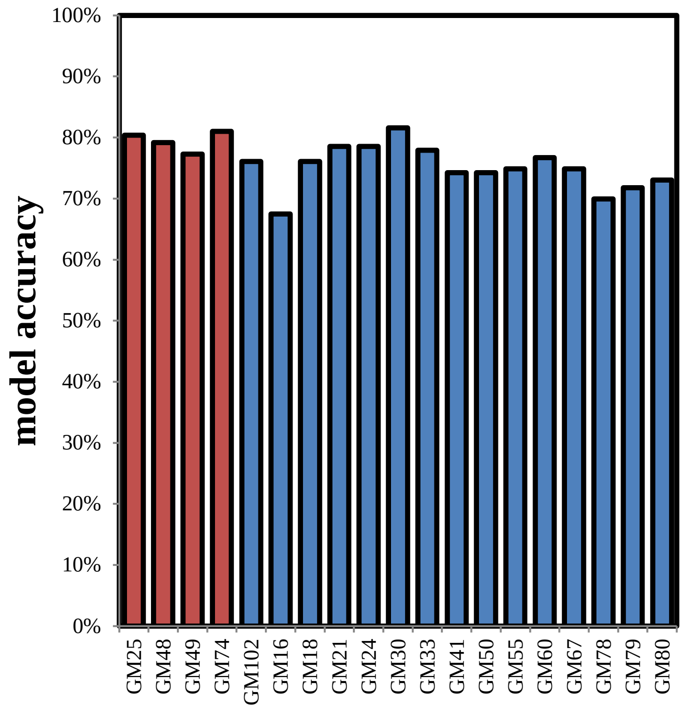


**Figure S2: Model accuracy.** Model accuracy defined as TP + TN over total predictions.

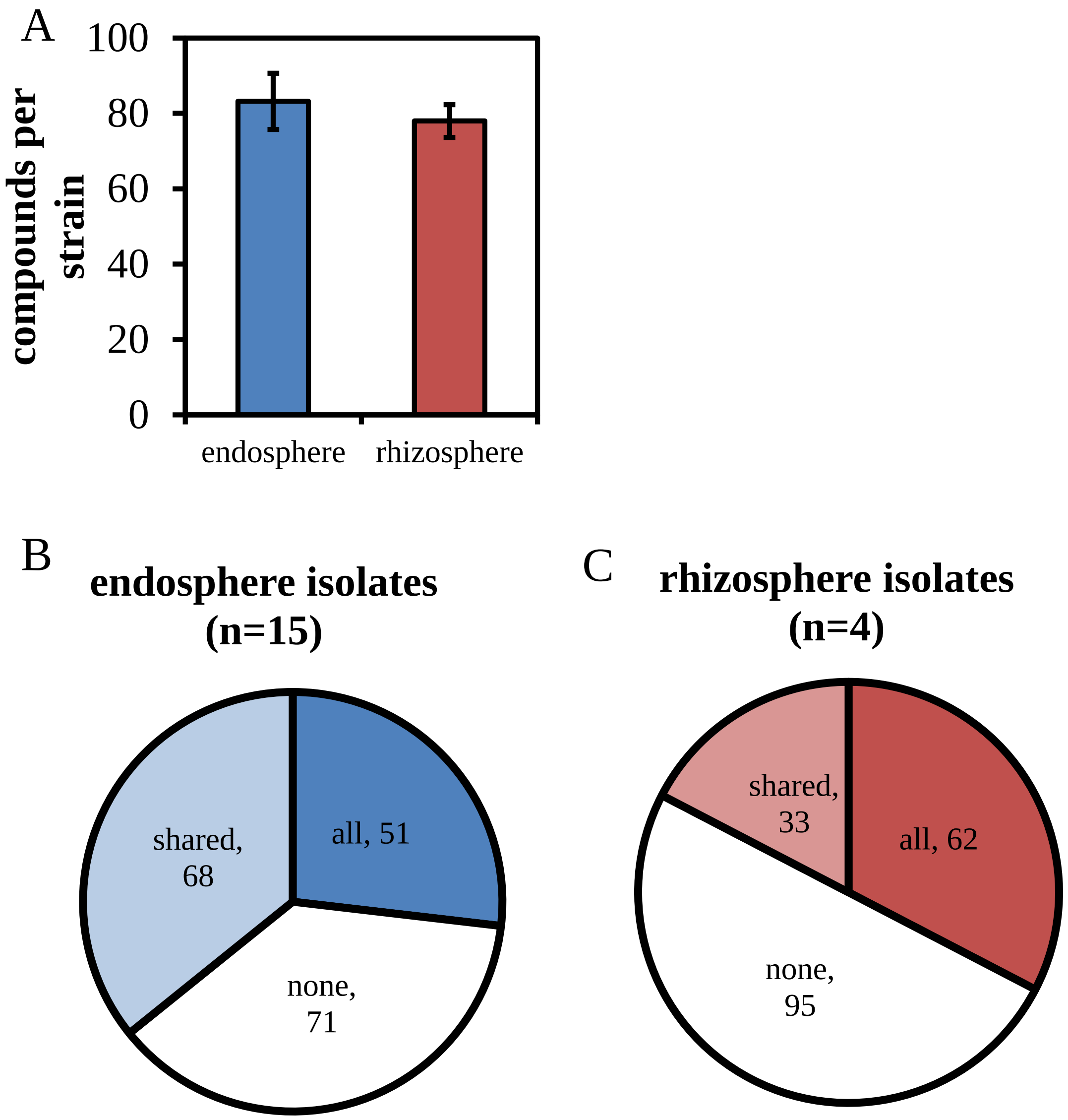


**Figure S3: Carbon substrate utilization summary for 190 tested compounds.** A) Bar chart showing average number of compounds utilized per strain, error bars are one standard deviation B) endosphere isolates C) rhizosphere isolates

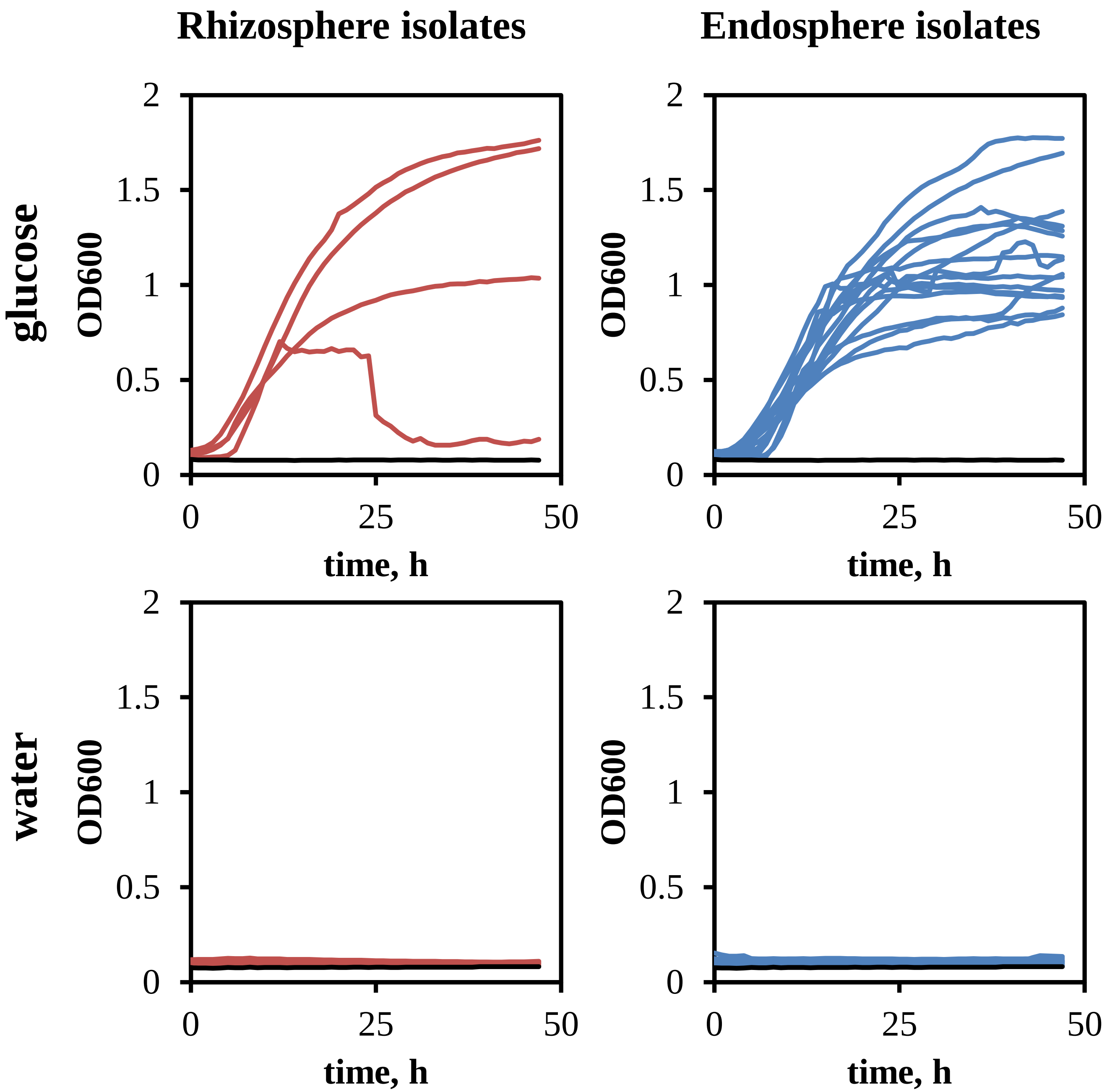


**Figure S4: Controls for M9 minimal media growth.** To accompany Figure 5D
