# Supplementary material for: Metabolic functions of Pseudomonas fluorescens strains from Populus deltoides depend on rhizosphere or endosphere isolation compartment
Source: Front Microbiol. 2015 Oct 14;6:1118. doi: 10.3389/fmicb.2015.01118 (PMC4604316; doi:10.3389/fmicb.2015.01118)

Supplemental File 1

Arabidopsis thaliana seedling growth in the presence of *Pseudomonas* isolate strains. Plants were grown on MS + 1% sucrose, then transferred to new plates and streaked with bacteria. Plants grown in the absence of bacteria were used as controls. Plant root growth or inhibition was assessed visually. The following slides show plants paired with respective control plants.

Control

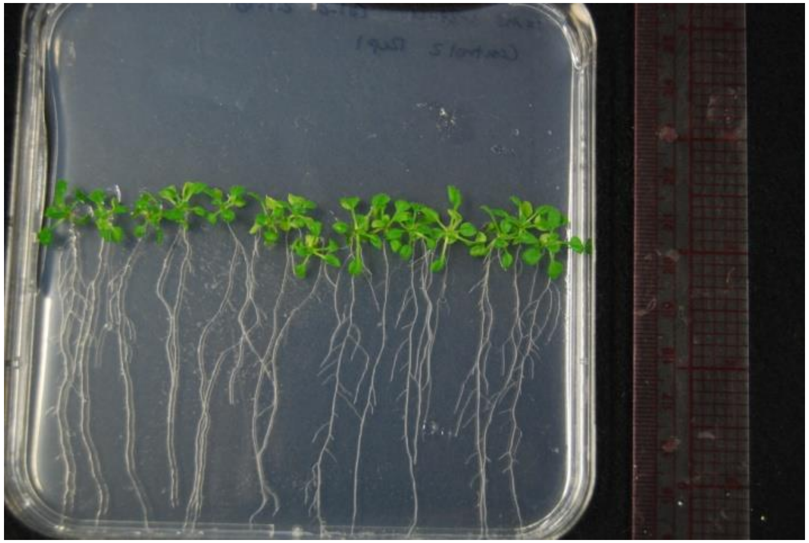

GM25

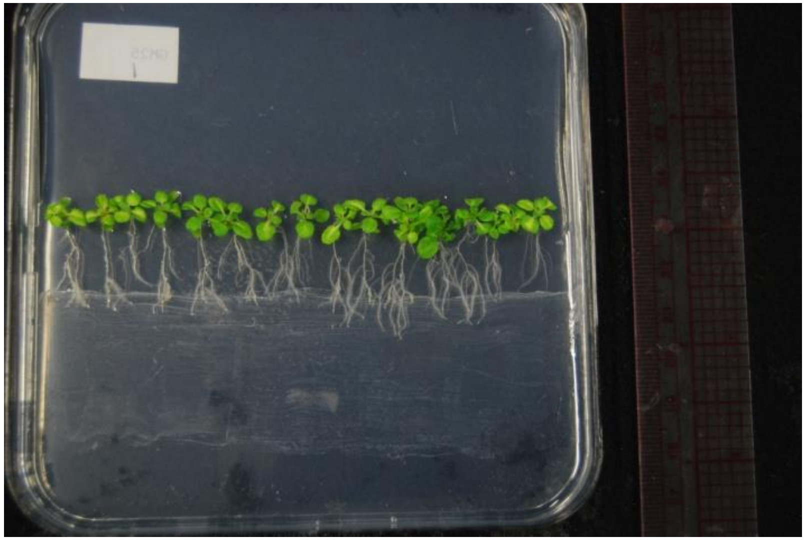

Control

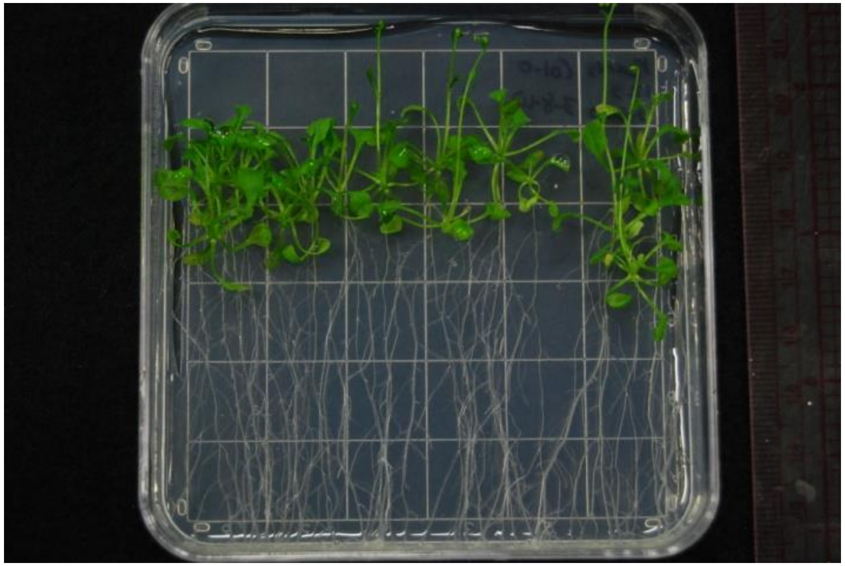

GM48

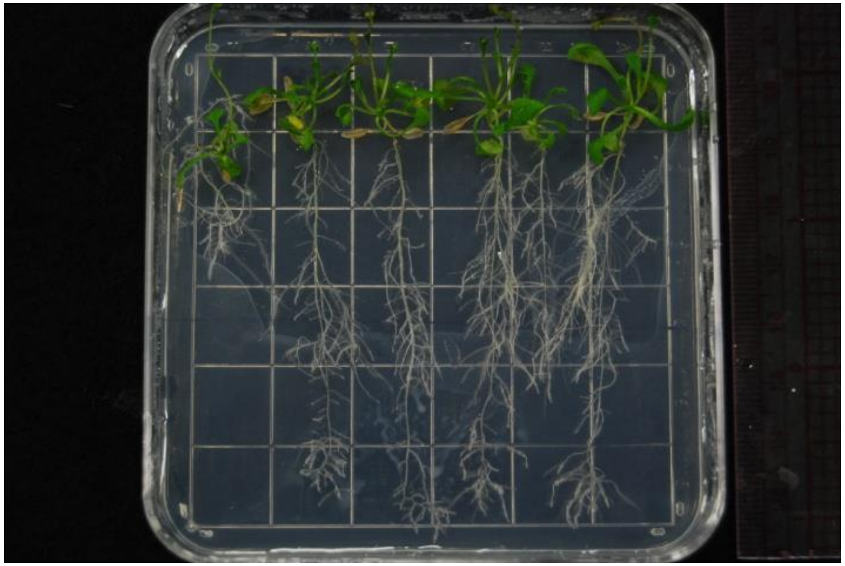

Control

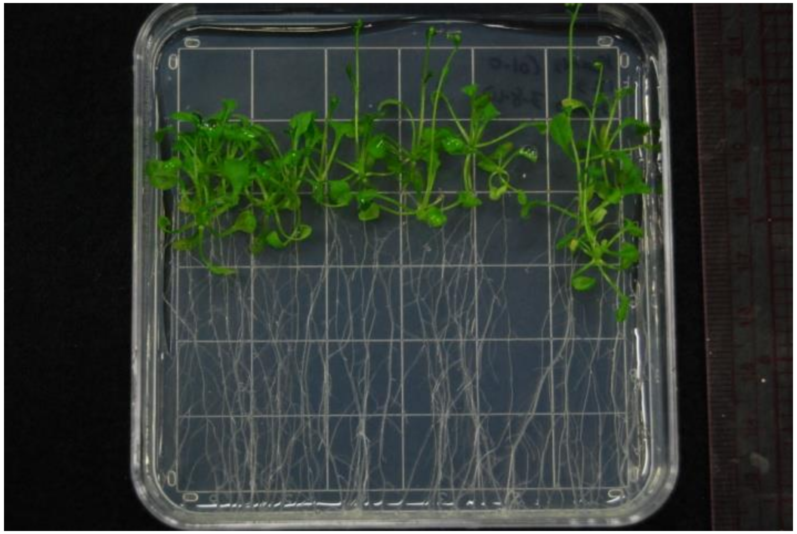

GM49

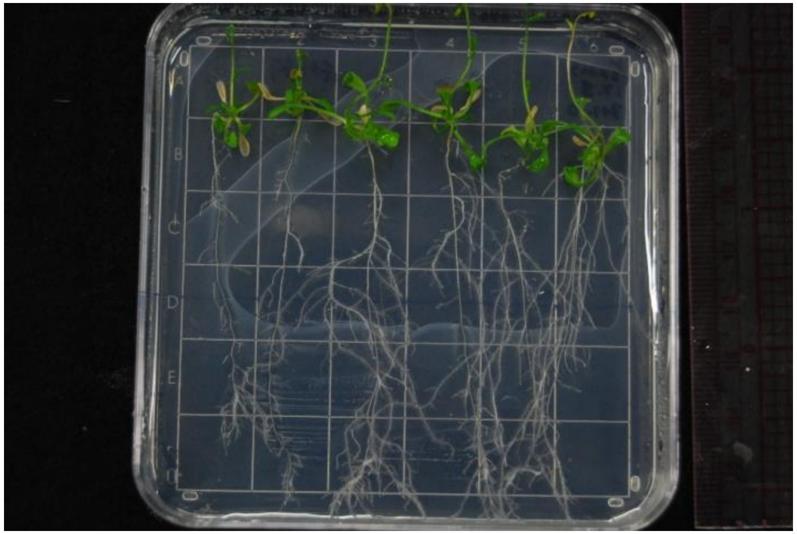

Control

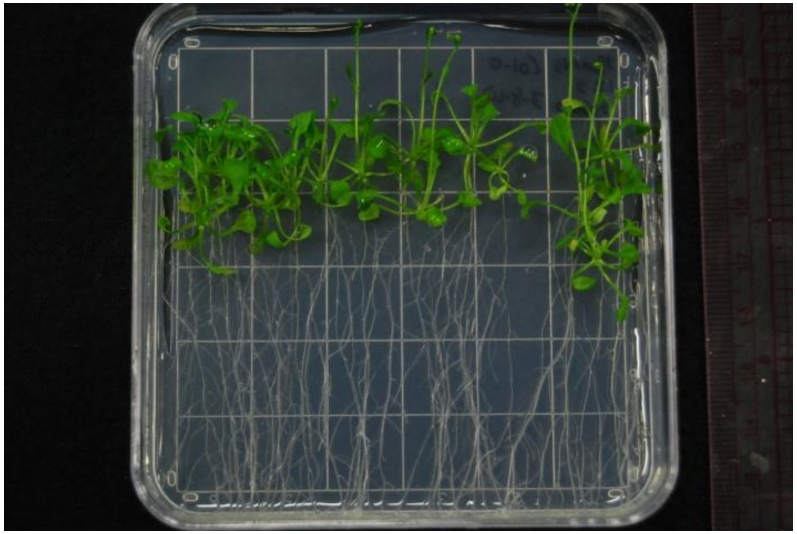

GM74

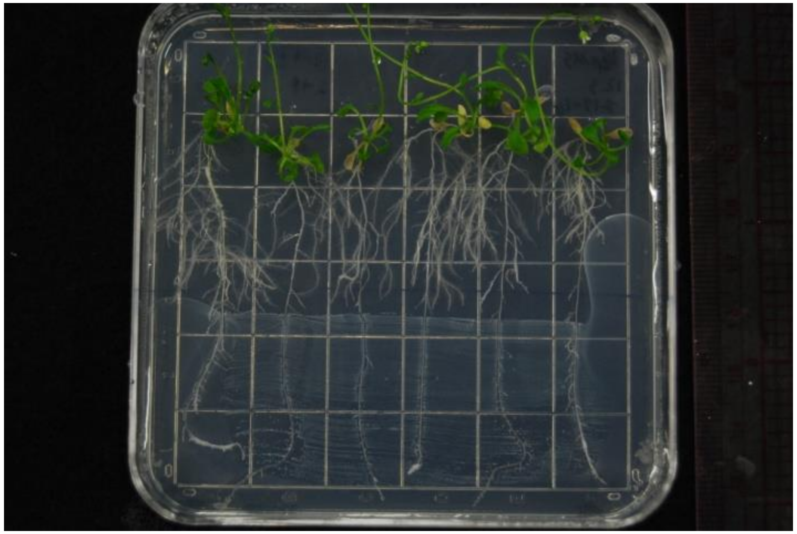

Control

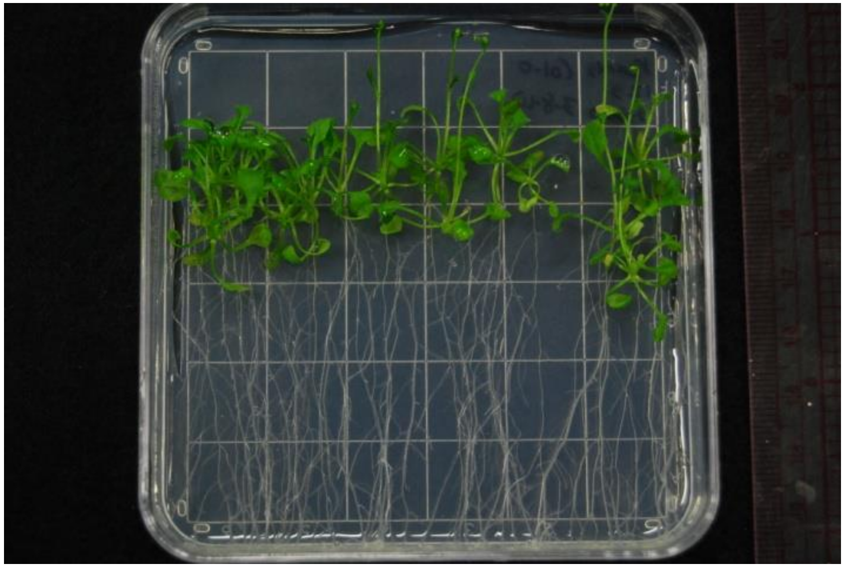

GM16

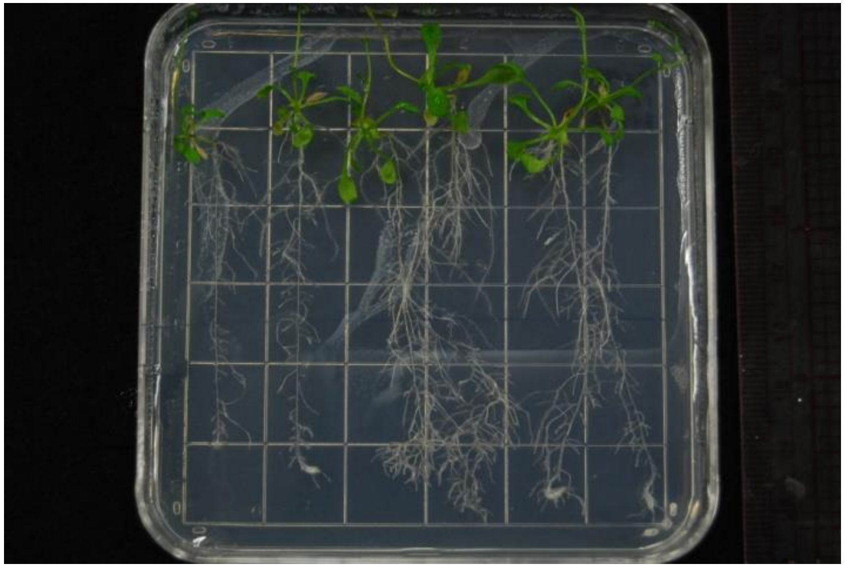

Control

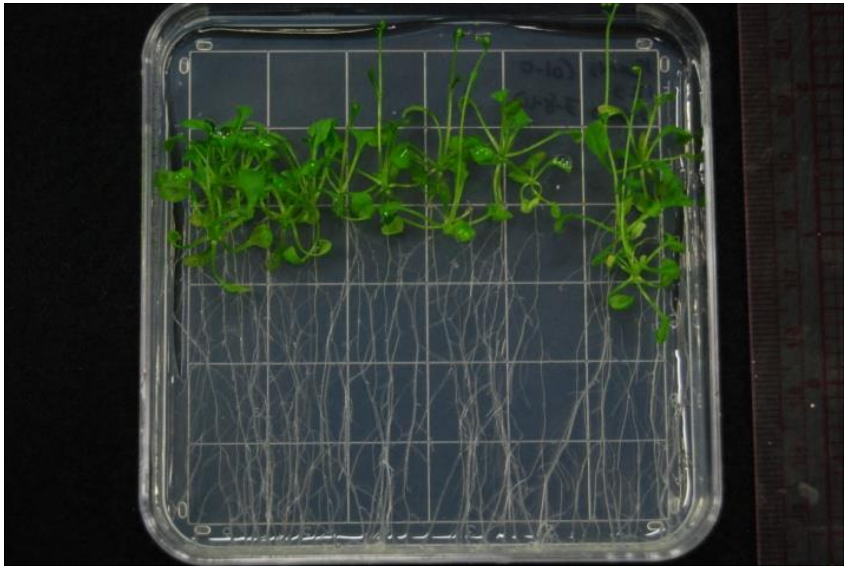

GM18

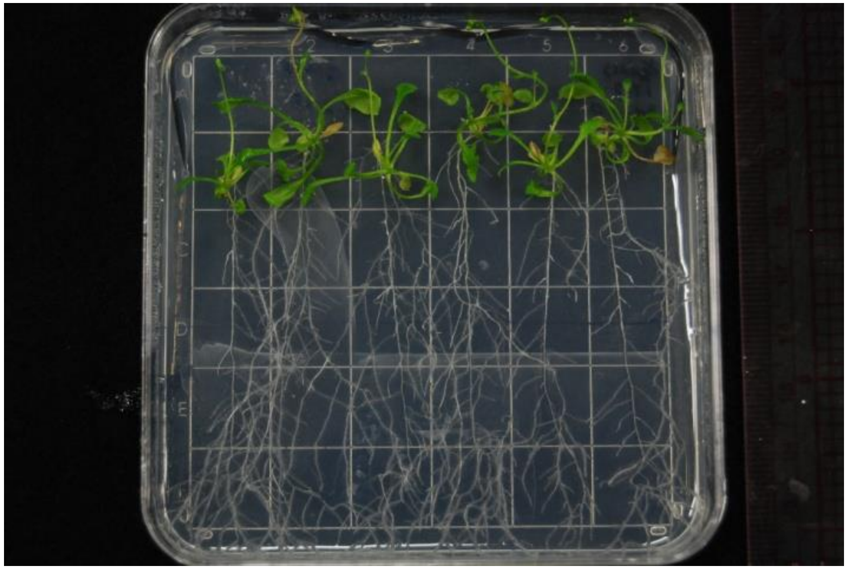

Control

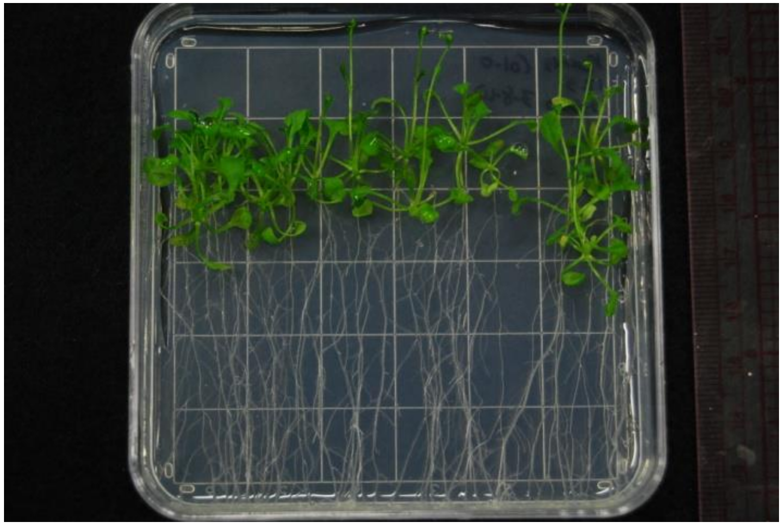

GM21

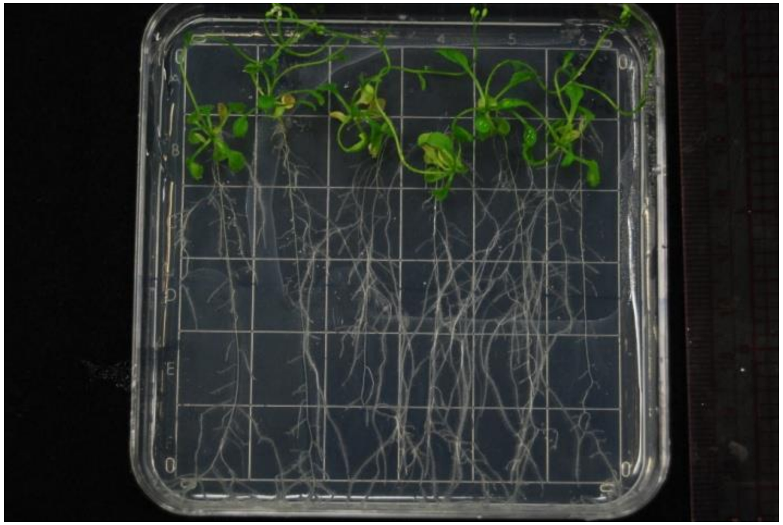

Control

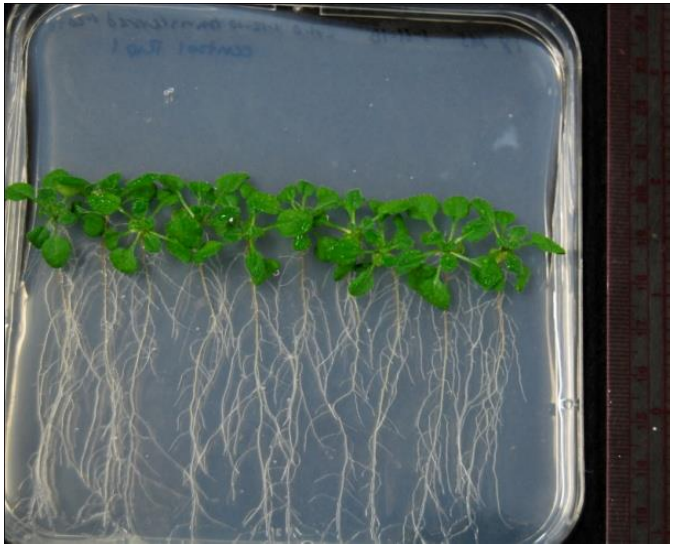

GM24

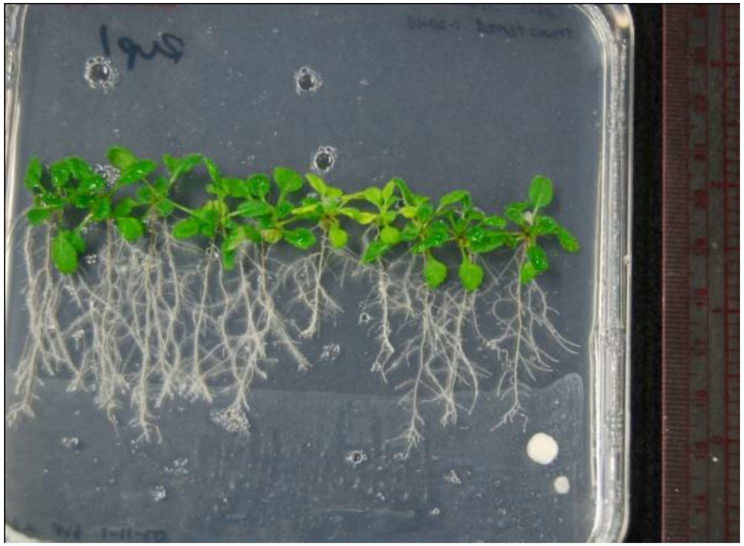

Control

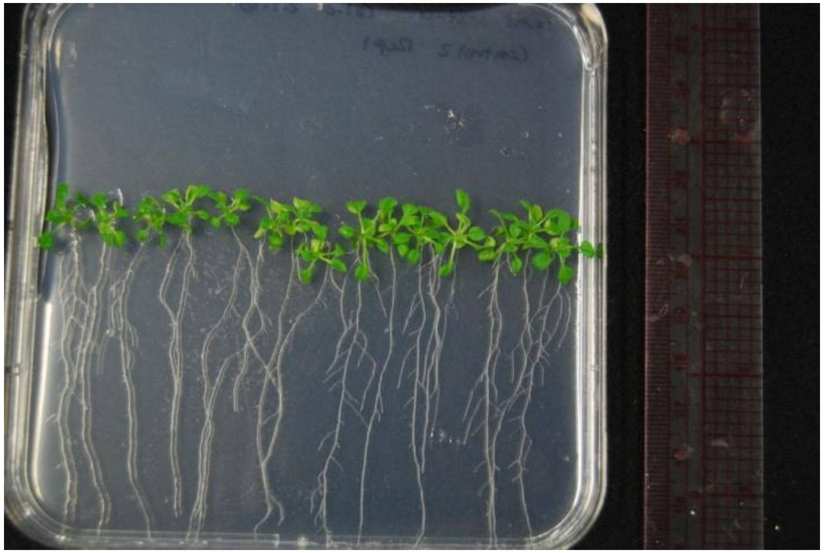

GM30

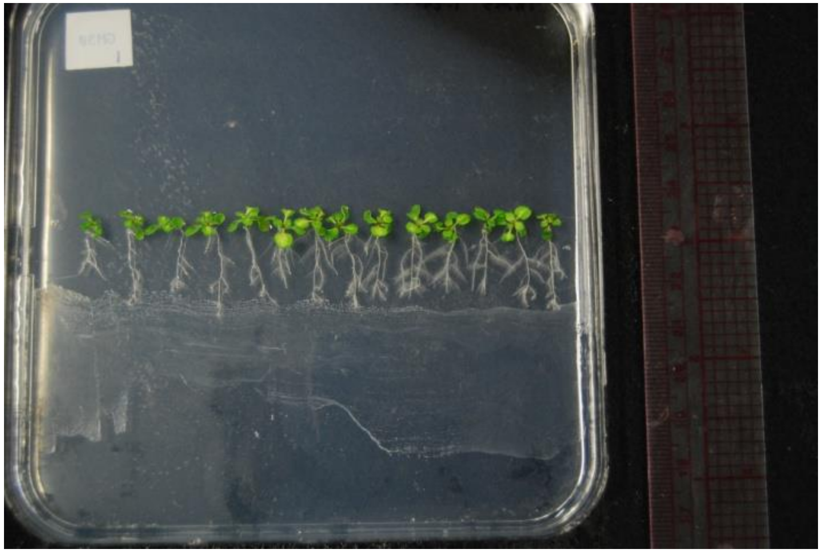

Control

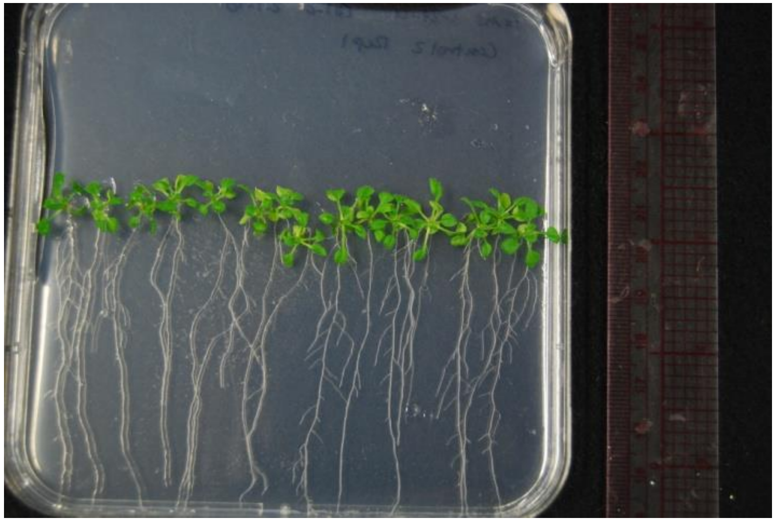

GM33

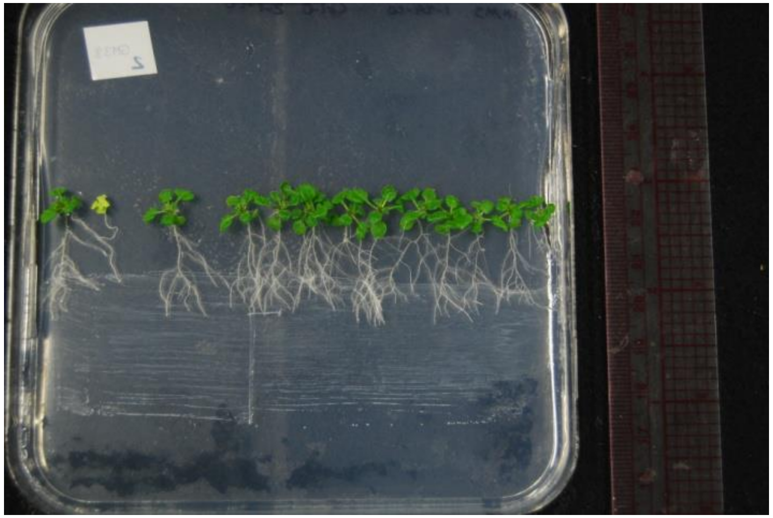

Control

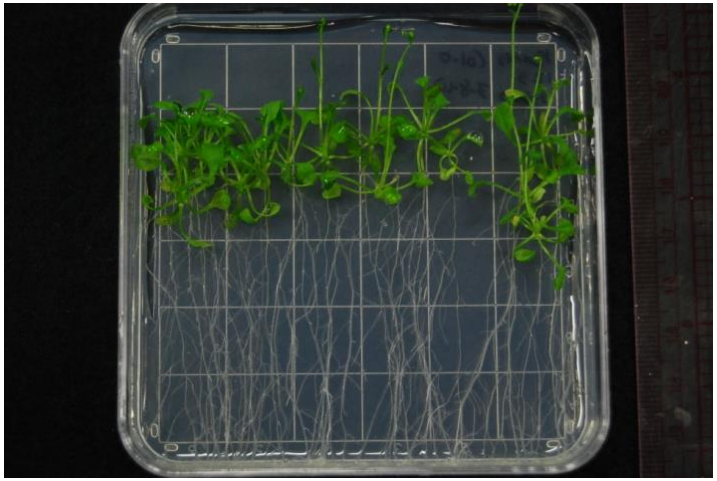

GM41

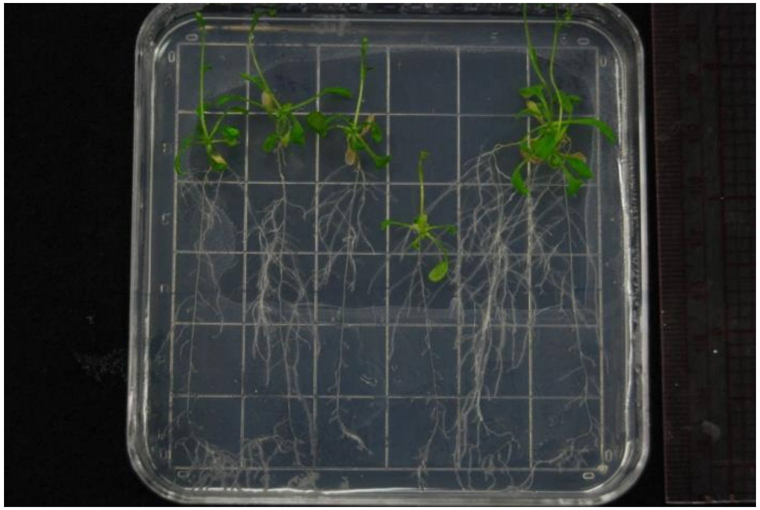

Control

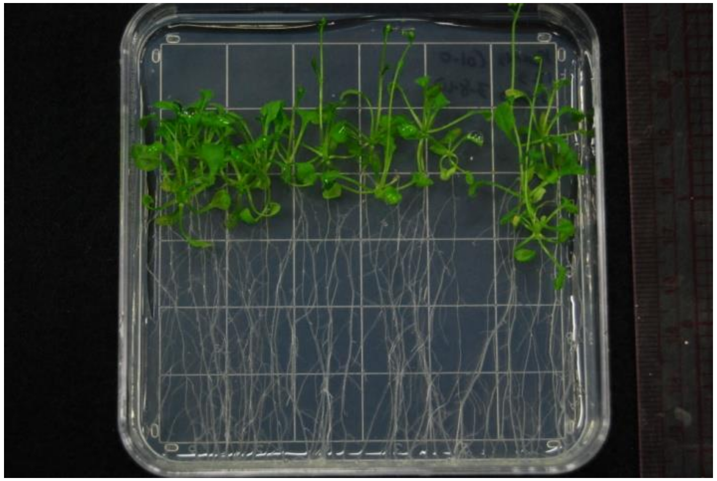

GM50

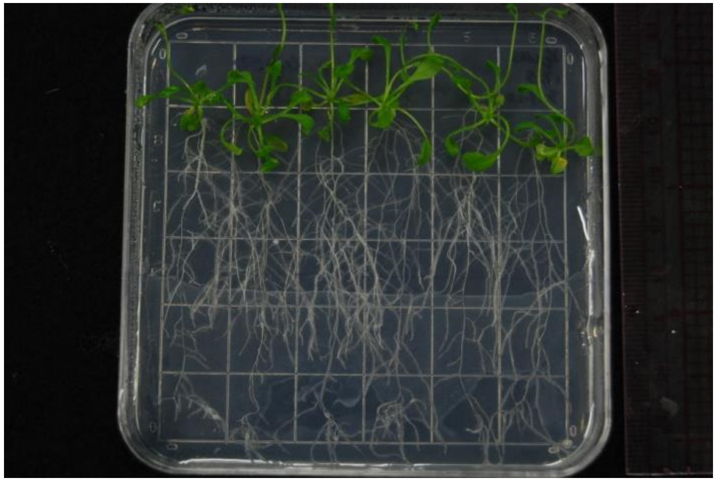

Control

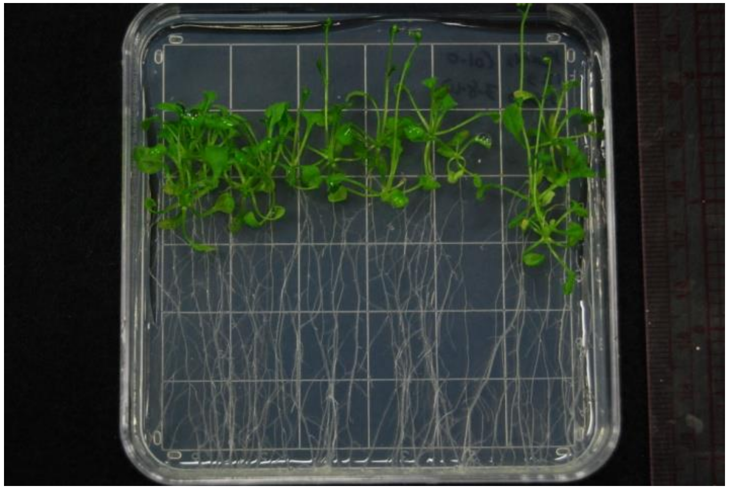

GM55

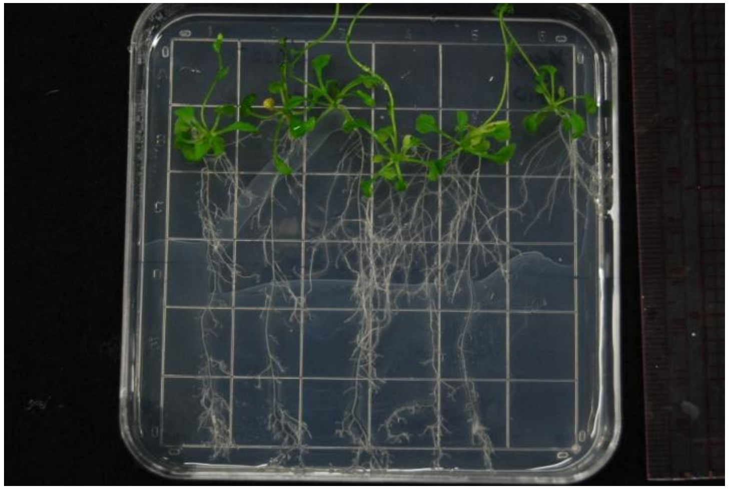

Control

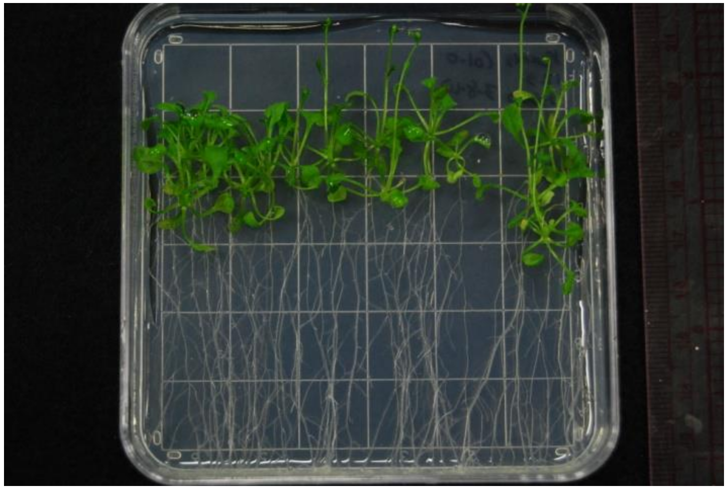

GM60

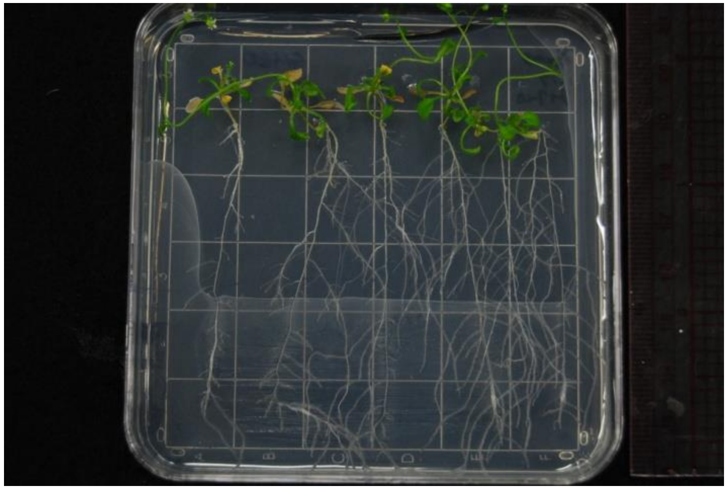

Control

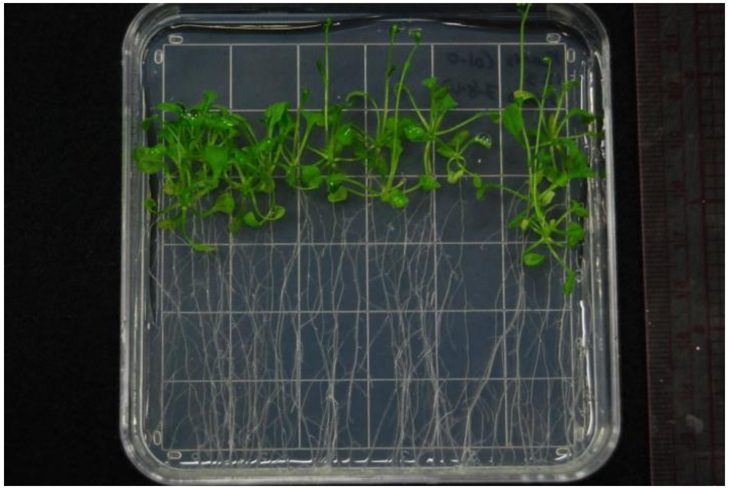

GM67

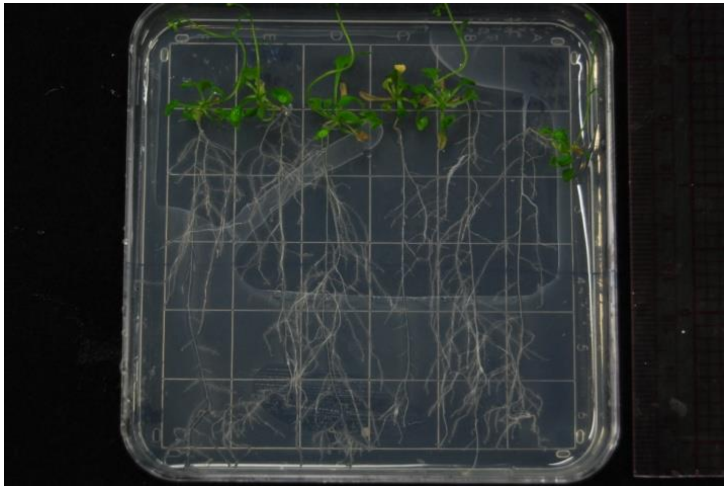

Control

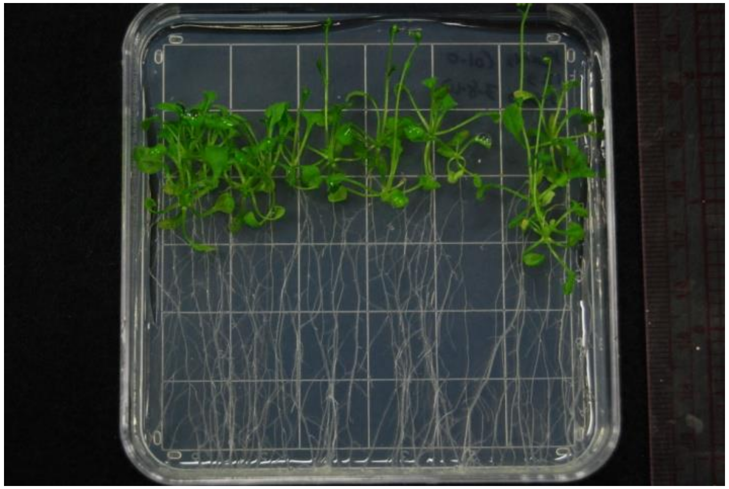

GM78

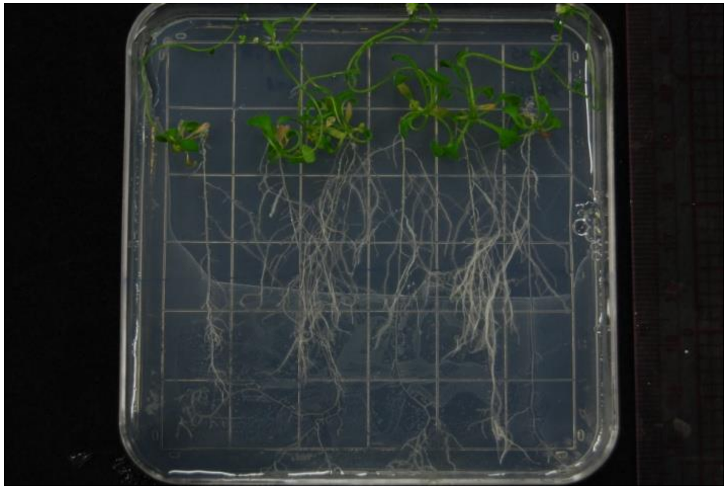

Control

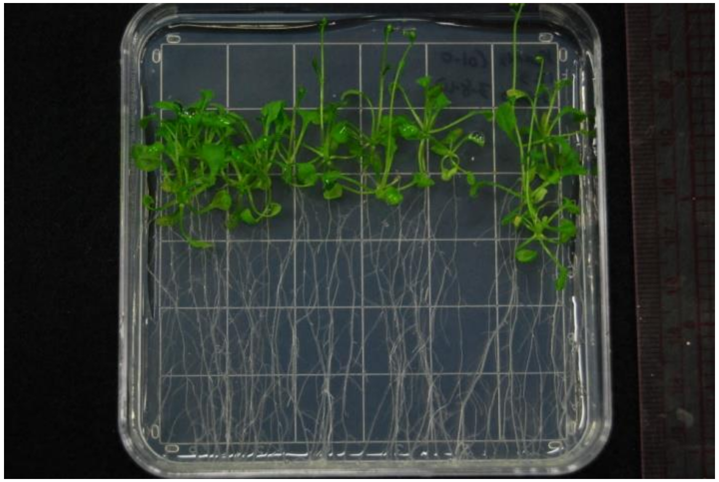

GM79

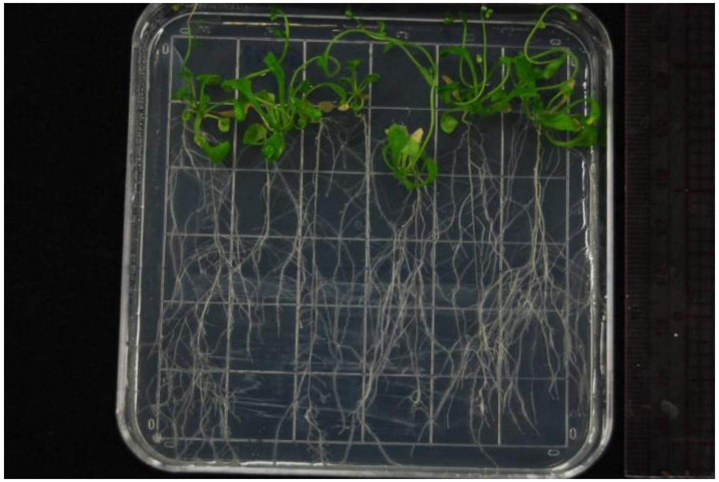

Control

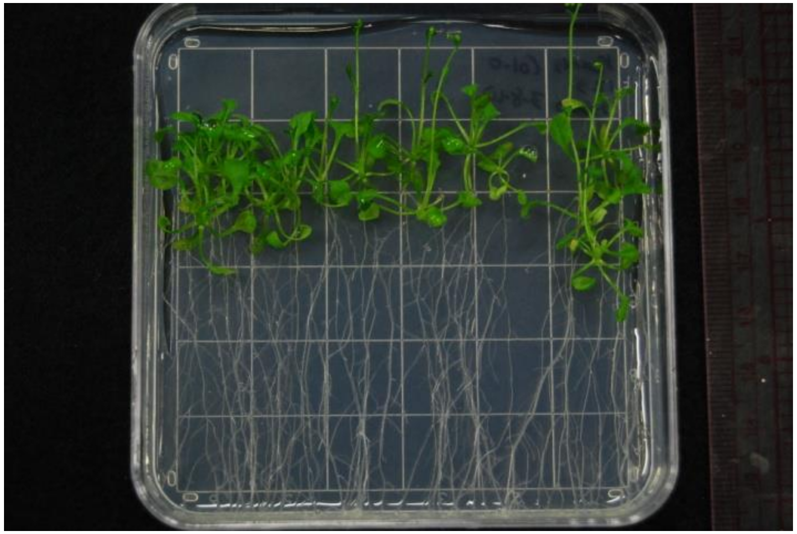

GM80

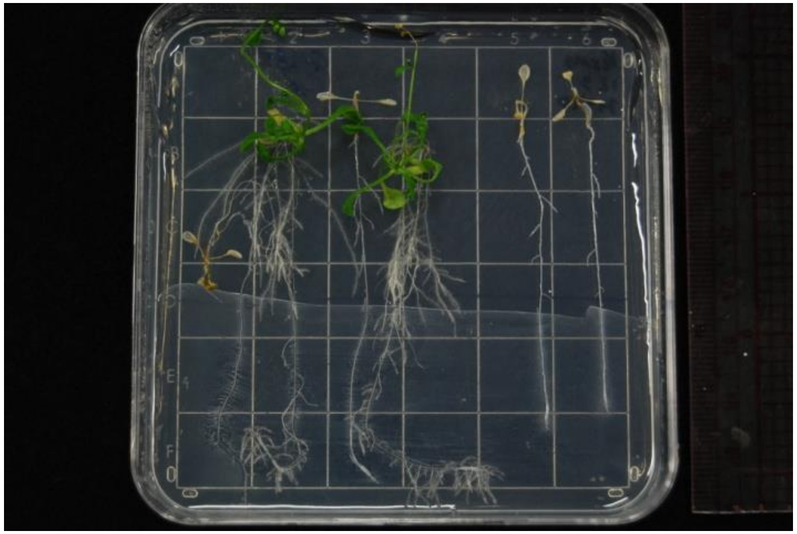

Control

GM102

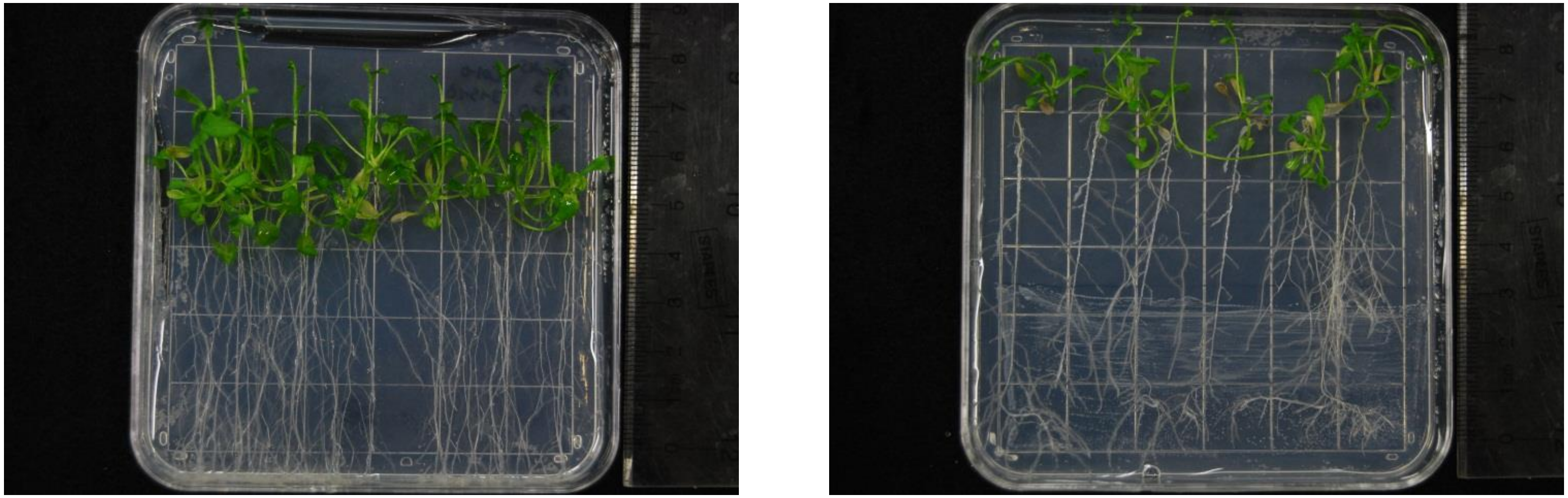

Supplement: Supplementary file 2 [file Image1.PDF]
